# Neural-Dynamic Based Synchronous-Optimization Scheme of Dual Redundant Robot Manipulators

**DOI:** 10.3389/fnbot.2018.00073

**Published:** 2018-11-08

**Authors:** Zhijun Zhang, Qiongyi Zhou, Weisen Fan

**Affiliations:** School of Automation Science and Engineering, South China University of Technology, Guangzhou, China

**Keywords:** dual-redundant-manipulators, redundant robot, complex tasks, motion planning, acceleration-level, neural dynamic method

## Abstract

In order to track complex-path tasks in three dimensional space without joint-drifts, a neural-dynamic based synchronous-optimization (NDSO) scheme of dual redundant robot manipulators is proposed and developed. To do so, an acceleration-level repetitive motion planning optimization criterion is derived by the neural-dynamic method twice. Position and velocity feedbacks are taken into account to decrease the errors. Considering the joint-angle, joint-velocity, and joint-acceleration limits, the redundancy resolution problem of the left and right arms are formulated as two quadratic programming problems subject to equality constraints and three bound constraints. The two quadratic programming schemes of the left and right arms are then integrated into a standard quadratic programming problem constrained by an equality constraint and a bound constraint. As a real-time solver, a linear variational inequalities-based primal-dual neural network (LVI-PDNN) is used to solve the quadratic programming problem. Finally, the simulation section contains experiments of the execution of three complex tasks including a couple task, the comparison with pseudo-inverse method and robustness verification. Simulation results verify the efficacy and accuracy of the proposed NDSO scheme.

## 1. Introduction

Redundancy resolution problem is an important issue in the control of redundant robot manipulators. The redundancy of the robot manipulators endows us with extra degrees-of-freedom to finish some subtasks in addition to the end-effector main task (Jin and Li, 2016; Reynoso-Mora et al., 2016; Guo et al., 2017; Huang et al., 2017). Control of dual-redundant-manipulators is more complex because they have twice degrees-of-freedom than a single-redundant manipulator does. With more and redundant degrees-of-freedom, dual-redundant-manipulators can not only complete the main task of the end-effectors, but also finish various subtasks, such as joint-limitation avoidance, obstacle avoidance, singularity avoidance, and dual-arms cooperations (Zhang et al., 2014; Liu et al., 2015; Jin et al., 2017; Chikhaoui et al., 2018).

For each manipulator of the dual-redundant-robot-manipulators, since the number *n* of degrees-of-freedom of joints is greater than the dimension *m* of end-effectors' position and posture, solutions to the inverse kinematic problem of each manipulator as same as dual-manipulators are infinite (i.e., the multiple-solution problem). In order to solve such a multiple-solution problem, a number of methods have been proposed (Chevallereau and Khalil, 1988; Jin and Zhang, 2014; Toshani and Farrokhi, 2014; Luo et al., 2017). The conventional method is the pseudo-inverse formulation $\dot{\theta }=J^{+}ṙ+\left(I-J^{+}J\right)z_{v}$ or $\ddot{\theta }=J^{+}\left(\ddot{r}-\overset{{}^{\circ}}{J}\dot{\theta }\right)+\left(I-J^{+}J\right)z_{a}$, which contains a specific minimum-norm solution plus a homogeneous solution (Lin and Zhang, 2013). The pseudo-inverse method has a simple form and has been applied to dual-redundant-manipulators (Zheng and Luh, 1986), but it has to compute the matrix inverse which may have high computational cost (Ho et al., 2005), algorithm singularities and have difficulty in containing $z_{v},z_{a}\in R^{n}$ into inequality form. That is to say, it cannot solve inequality constrain problems (Cheng et al., 1994). What's worse, the determining the magnitude of *z*_*v*_ and *z*_*a*_ is based on trial-and-error approach and is over-dependent on subjective judgement and experience (Zhang et al., 2004). Although some improved pseudo-inverse methods have been developed in recent years, such as joint torque optimization (Flacco and De Luca, 2015; Wang et al., 2015; Xiao et al., 2016), but it still cannot solve the inequality problems.

A repetitive motion is a basic requirement of redundant-robot-manipulators in practical applications if they are expected to execute cyclic tasks. A repetitive motion is that when the end-effector tracks a closed path in Cartesian space, all the joint trajectories should be closed. That is to say, the final states of joints must coincide with the initial ones when the end-effector completes a closed end-effector path. If this issue is not considered into the motion planning scheme of dual-redundant-manipulators, the joint-drift phenomenon would happen. In order to realize repetitive motions, additional self-motion strategy is necessary to readjust the joints of dual-manipulators to the initial states at the end of each cycle. Evidently, this is much inefficient and is not acceptable in a factory automation assembly line. Klein firstly studied this problem in a single redundant-robot-manipulator, and his research showed that the joint-drift that occurred in the pseudo-inverse control scheme is not unpredictable (Klein and Kee, 1989). In the last two decades, in order to solve the joint-drift problem, many quadratic-programming-based repetitive motion planning schemes have been proposed and solved by neural networks but most of them are about the single redundant robot manipulator (Zhang et al., 2008, 2018; Zhang and Zhang, 2012, 2013b). The control methodology of dual-redundant manipulators is imperative, as there are more and more complex end-effector tasks, such as unscrewing caps (Felip and Morales, 2015), grasping and moving of an object (Shin and Kim, 2015; Dong et al., 2017). These tasks can not be completed by a single manipulator and need dual-robot-manipulators. In recent years, some researchers have proposed impedance and admittance control methods to dual-arms coordination. For example, Lee et al. (2014) and Jr and Roberts (2015) proposed a novel relative impedance control based on the relative Jacobian expression. These works more focus on dual-arms cooperation and allocating task through force/torque, and the force/torque sensors are necessary. In fact, some tasks only need dual-manipulators synchronous working and cooperation. For instance, moving a heavy box. To finish these tasks, some researchers exploited quadratic-programming-based repetitive motion planning scheme for dual-redundant-manipulators and then used neural network as a quadratic programming solver. In our previous work, a neural dynamic method based repetitive motion planning scheme was proposed for humanoid robot arms (Zhang et al., 2015), but it is on velocity-level and cannot consider the joint-acceleration limits. In addition, the velocity-level repetitive motion planning scheme can not be directly applied to acceleration controlled robots. Jin and Zhang proposed a repetitive motion planning scheme at acceleration level (Jin and Zhang, 2014). However, the scheme is only performed on dual-manipulators with simple planar three links, and the end-effector tasks are very simple. It is worth pointing out that very few acceleration-level repetitive motion planning schemes take position-error feedback into consideration to make the position-error convergent as time involves.

The studying motivations of this paper can be summarized as: 1) A repetitive motion is a basic requirement of redundant-robot-manipulators in practical applications. 2) Most researches on the repetitive motion planning are based on a single-manipulator with less degrees-of-freedom, and very few researches considered the synchronous-optimization scheme of dual redundant robot manipulators. 3) The traditional resolution scheme at the velocity level cannot consider the acceleration limit avoidance, which may lead to acceleration limitation exceeded problem. In order to resolve the redundancy problem of dual-redundant-robot-manipulators with 14 degrees-of-freedom, a neural-dynamic based synchronous-optimization scheme of dual redundant robot manipulators (NDSO) is proposed in this paper. Different from the existing work (Jin and Zhang, 2014), the proposed NDSO scheme can be performed on dual-redundant-manipulators with 14 degrees-of-freedom and working in three-dimensional space. In addition, the dual-redundant-manipulators can track some complex paths (such as geometric curves and numbers) and complete coupled tracking task. Furthermore, the NDSO scheme has excellent robustness under the perturbation of systematic error.

The remainder of the paper is organized into four sections. In section 2, the neural-dynamic based synchronous-optimization subschemes (Sub-NDSO) of the left and right manipulators are formulated. In section 3, the Sub-NDSO of the left and right manipulators are unified into a standard quadratic programming problem, which is equivalent to a piecewise-linear projection equation, and then solved by a linear variational inequalities-based primal-dual neural network (LVI-PDNN). Section 4 shows the simulation result that the NDSO scheme performed on dual-redundant-manipulators to track three complex end-effector tasks in three-dimensional space. Comparison experiments and robustness verification experiment with perturbed LVI-PDNN are also conducted and the related results are showed in this section. Section 5 concludes this paper with final remarks.

The main contributions of the paper are as follows.

1. A neural-dynamic based synchronous-optimization scheme of dual redundant robot manipulators (NDSO) is proposed to solve the joint-drift phenomena at the joint-acceleration level. The advantage of the NDSO scheme is that it can not only complete the traditional end-effector tasks but also some couple tasks. In addition, the physical limit constraints allow the scheme to apply to actual situations because it guarantees the robot joints not to exceed their physical limits. In addition, it is easier than velocity-level scheme to conduct such a scheme on an acceleration/torque controlled manipulator.
2. The NDSO scheme works for dual-robot-manipulator system, which has twice degrees-of-freedom than the same model single-robot-manipulator and thus has better coordination and flexibility compared with a single robot manipulator. Evidently, the dual-redundant-manipulator with the NDSO scheme can complete more complex and heavy tasks. It is convenient to make adjustment to the original scheme through changing the definition of matrixes in order to achieve better results because the scheme is based on a standard quadratic programming form.
3. Three complex end-effector tasks, i.e., a pentagram tracking, a number “47” writing and a couple task, are completed by three-dimensional dual-redundant-manipulators, which validate the efficiency and accuracy of the proposed NDSO scheme.
4. The simulation experiment verifies the robustness of the NDSO scheme with the perturbation of the systematic error. That means the proposed scheme will have strong capacity of anti-disturbance considering practical scenarios.

**Figure 1:**
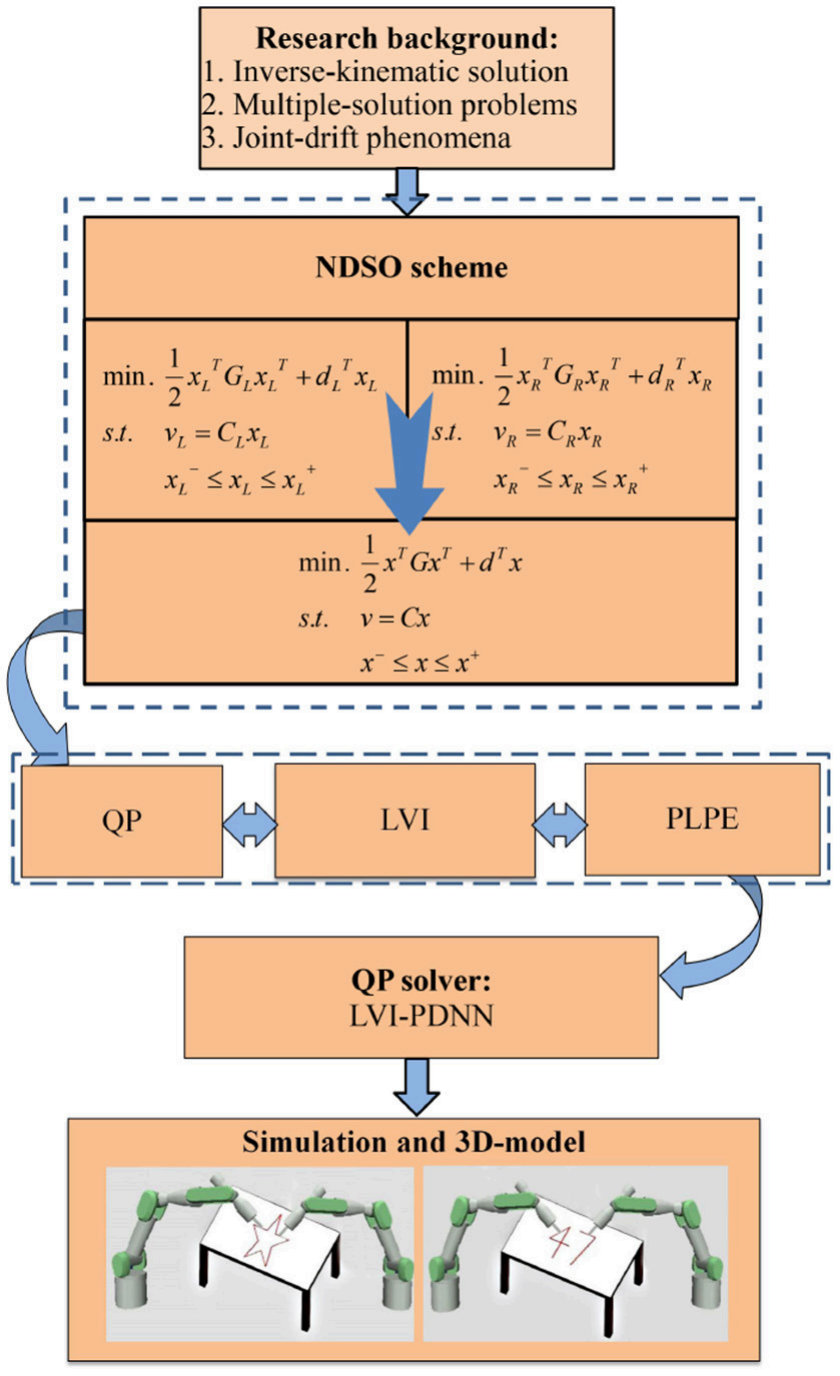
System structure of the neural-dynamic based synchronous-optimization scheme of dual redundant robot manipulators (NDSO). It visualizes the logical structure of the paper starting from background analysis, then the problem formulation and finally the simulation.

Before ending this section, the system structure of the scheme can be seen from Figure 1. First of all, the performance indices of the left and right arms are obtained by using neural dynamic method twice. Next, considering the position and velocity error, joint-angle, joint-velocity and joint-acceleration limits, the repetitive motion planning subschemes of left and right arms are constructed. Furthermore, by combining the repetitive motion planning subschemes of left and right arms, the NDSO scheme is obtained, which is further unified into a standard quadratic programming problem. The quadratic programming problem (i.e., QP in the figure) is equivalent to a set of linear variational inequalities problem (i.e., LVI in the figure) and is finally equivalent to a piecewise linear projection equation (i.e., PLPE in the figure). Finally, the piecewise linear projection equation is solved by a linear variational inequalities-based primal-dual neural network (LVI-PDNN).

## 2. Problem formulation

In this section, a forward kinematic equation is first presented. Next, an acceleration-level feedback is designed. Third, an acceleration-level repetitive motion criterion is deduced by neural dynamic method two times.

### 2.1. Preliminaries

For simplicity, we use the subscript L/R to represent the left and right redundant manipulators. The kinematic equations of the left or right arm of the dual-redundant-manipulators at position level, velocity level and acceleration level are formulated respectively as

(1) $$f_{\text{L/R}}(\theta_{\text{L/R}})=r_{\text{L/R}}(t)$$

(2) $$J_{\text{L/R}}(\theta_{\text{L/R}}){\dot{\theta }}_{\text{L/R}}(t)={\dot{r}}_{\text{L/R}}(t)$$

(3) $$J_{\text{L/R}}(\theta_{\text{L/R}}){\ddot{\theta }}_{\text{L/R}}(t)={\ddot{r}}_{\text{aL/R}}(t)={\ddot{r}}_{\text{L/R}}(t)-{\dot{J}}_{\text{L/R}}(\theta_{\text{L/R}}){\dot{\theta }}_{\text{L/R}}(t)$$

where $r_{\text{L/R}}\left(t\right),{ṙ}_{\text{L/R}}\left(t\right),\text{and}\;{\ddot{r}}_{\text{L/R}}\left(t\right)\in R^{m}$ denote the position-and-orientation vector, velocity vector, and acceleration vector of an end-effector, θ_L/R_(*t*), ${\dot{\theta }}_{\text{L/R}}\left(t\right),\text{and}\;{\ddot{\theta }}_{\text{L/R}}\left(t\right)\in R^{n}$ denote the joint angle, joint velocity and joint acceleration of the left or right manipulator, Jacobian matrix *J*_L/R_(θ_L/R_) = ∂*f*_L/R_(θ_L/R_)/∂θ_L/R_, matrix ${\dot{J}}_{\text{L/R}}\left(\theta_{\text{L/R}}\right)$ is the first order derivation of Jacobian matrix *J*_L/R_(θ_L/R_) with respect to time *t*. In this paper, since one manipulator has seven degrees-of-freedom and the task is performed in a three dimensional space, *n* = 7 and *m* = 3. In Equation (1), θ_L/R_(*t*) and *r*_L/R_(*t*) are related via a nonlinear function *f*_L/R_(·). If θ_L/R_(*t*) is known, it is easy to compute *r*_L/R_(*t*) since *f*_L/R_(·) can be uniquely determined by a given redundant robot manipulator. This process is called a forward kinematic resolution. On the contrary, it is very difficult to compute directly θ_L/R_(*t*) if *r*_L/R_(*t*) is known because it is difficult to obtain the inverse function $f_{\text{L/R}}^{-1}\left(\cdot \right)$ of nonlinear function *f*_L/R_(·). That is to say, an inverse kinematic problem of a redundant robot manipulator (or termed redundancy problem) is a difficult problem.

**Remark:** In practical systems, the control inputs are sometimes subject to the saturation problem and uncertainties. Many methods have been proposed to solve the issues such as (Tran et al., 2015; Eremin and Shelenok, 2017; Sun et al., 2017, 2018). Since we only focus on the redundancy resolution problem and it is assumed that the control inputs satisfy the condition, the saturation problem and uncertainties are out of our research scope, and are ignored here.

### 2.2. Acceleration-level forward equation with feedback

In practical applications, error feedback should be considered in Equation (3). With the following theorem, the acceleration-level forward equation with feedback is obtained, i.e.,v

**Theorem 1**. *Considering an end-effector motion of a robot manipulator, for any scalar parameters ρ_V_ > 0 and ρ_P_ > 0, the error-feedback included acceleration-level forward kinematic equation is*

(4) $$\begin{array}{l} J(\theta )\ddot{\theta }(t)={\ddot{r}}_{d}(t)-\dot{J}(\theta )\dot{\theta }(t)+\rho_{V}({\dot{r}}_{d}(t)-J(\theta )\dot{\theta }(t))+\rho_{P}(r_{d}(t)\\ \;-f(\theta )), \end{array}$$

where *r*_d_, ṙ_d_, and ${\ddot{r}}_{\text{d}}$ denote desired end-effector path, desired end-effector velocity, and desired end-effector acceleration, respectively; θ, $\dot{\theta }$, and $\ddot{\theta }$ denote the joint-angular variable, joint-velocity variable, and joint-acceleration variable; Function *f*(θ) is a continuous nonlinear mapping function with known parameters for a given robot; *J*(θ) and $\dot{J}\left(\theta \right)$ are the Jacobian matrix and the first order derivative of Jacobian matrix; parameters ρ_V_ > 0 and ρ_P_ > 0 are the feedback coefficients of velocity and position errors, respectively. With these error feedbacks, the end-effector position error would converge exponentially to zero.

***Proof 1***. Considering the following state-equations of two dimensional linear system

(5) $$\dot{\chi }(t)=A\chi (t),$$

(6) y(t)=Qχ(t),

where $\chi \left(t\right)={\left[\chi_{1}\left(t\right),\chi_{2}\left(t\right)\right]}^{\text{T}}$ is the the state vector consisting of two state variables as its elements; $\dot{\chi }\left(t\right)={\left[{\dot{\chi }}_{1}\left(t\right),{\dot{\chi }}_{2}\left(t\right)\right]}^{\text{T}}$ is the time derivative of the state vector χ(*t*); *y*(*t*) = [*y*_1_(*t*)] is an output vector consisting of two outputs as its elements, and *A* and *Q* are the coefficient matrices.

In order to make the position error converge to zero at the end of each cycle, an error function *E*_*f*_(*t*) is defined as

(7) $$E_{f}(t)=r_{\text{dL/R}}(t)-f(\theta_{\text{L/R}})$$

where *r*_dL/R_(*t*) denotes the desired end-effector path. Its first-order and second-order derivative with time *t* (i.e., the velocity error Ė_*f*_ and acceleration error Ë_*f*_) are

(8) $${\dot{E}}_{f}(t)={\dot{r}}_{\text{dL/R}}(t)-J_{\text{L/R}}(\theta_{\text{L/R}}){\dot{\theta }}_{\text{L/R}}(t),$$

(9) $${\ddot{E}}_{f}(t)={\ddot{r}}_{\text{dL/R}}(t)-{\dot{J}}_{\text{L/R}}(\theta_{\text{L/R}}){\dot{\theta }}_{\text{L/R}}(t)-J_{\text{L/R}}(\theta_{\text{L/R}}){\ddot{\theta }}_{\text{L/R}}(t)$$

respectively.

For the convenience of analysis, the state variables χ_1_ and χ_2_ are set as *E*_*f*_ and Ė_*f*_, respectively, i.e.,

(10) $$\chi =\left[\begin{array}{c} E_{f}\\ {\dot{E}}_{f} \end{array}\right],\dot{\chi }=\left[\begin{array}{c} {\dot{E}}_{f}\\ {\ddot{E}}_{f} \end{array}\right].$$

**Figure 2:**
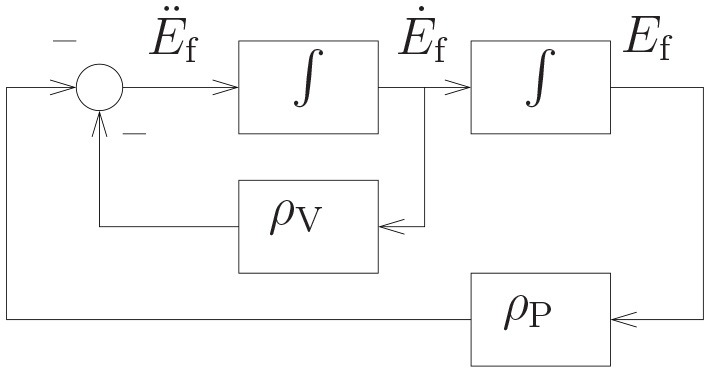
The simulation diagram of the position and velocity feedback.

In addition, by defining

$$A=\left[\begin{array}{cc} 0 & 1\\ -\rho_{\text{P}} & -\rho_{\text{V}} \end{array}\right]\;\text{and}\;Q=\left[\begin{array}{cc} 1 & 0 \end{array}\right],$$

with ρ_V_ > 0 and ρ_P_ > 0, the state-equations (5) and (6) are equivalent to the following second order differential equation

(11) $${\ddot{E}}_{f}=-\rho_{\text{V}}{\dot{E}}_{f}-\rho_{\text{P}}E_{f}$$

where ρ_V_ > 0 and ρ_P_ > 0 are the feedback coefficients of velocity and position errors, respectively. Figure 2 shows the simulation diagram of the position and velocity feedback based on Equation (11). Substituting (7)–(9) into (11), we obtain

(12) $$\begin{array}{l} J_{\text{L/R}}(\theta_{\text{L/R}}){\ddot{\theta }}_{\text{L/R}}(t)={\ddot{r}}_{\text{afL/R}}(t)={\ddot{r}}_{\text{dL/R}}(t)-{\dot{J}}_{\text{L/R}}(\theta_{\text{L/R}}){\dot{\theta }}_{\text{L/R}}(t)\\ \;+\rho_{\text{V}}({\dot{r}}_{\text{dL/R}}-J_{\text{L/R}}(\theta_{\text{L/R}}){\dot{\theta }}_{\text{L/R}}(t))+\rho_{\text{P}}(r_{\text{dL/R}}(t)-f(\theta_{\text{L/R}})). \end{array}$$

Equation (4) is thus proved.

Next, we will prove the exponential convergence performance of the position errors *E*_*f*_(*t*). According to modern control theory (Tewari, 2002), characteristic roots ϱ_1_ and ϱ_2_ of the system matrix *A* can be obtained by solving the following characteristic equation

(13) $$|\varrho I-A|=|\left[\begin{array}{cc} \varrho & -1\\ \rho_{\text{P}} & \varrho +\rho_{\text{V}} \end{array}\right]|=\varrho^{2}+\rho_{\text{V}}\varrho +\rho_{\text{P}}=0,$$

where *I* is an identity matrix, |·| is the determinant notation, and ϱ is the characteristic root of Equation (13), which is determined by the coefficients ρ_P_ and ρ_V_ of characteristic Equation (13).

Since the position error *E*_*f*_(*t*) and the velocity error Ė_*f*_(*t*) are the elements of state vector χ(*t*), discussion of the time-domain response of the state variables χ(*t*) is equivalent to discussion of errors. Based on modern control theory (Tewari, 2002), if the initial state χ(0) = χ_0_ is determined, the unique solution of the state-equation (5) can be represented as

(14) χ(t)=Φ(t)χ(0),

where Φ(*t*) = *e*^*At*^.

Considering *A* = [0, 1;−*ρ*_P_, −*ρ*_V_], based on characteristic Equation (13), the time-domain response of the state variables χ(*t*) (i.e., Equation 14) can be discussed according to the following three situations.

From the formula of root, we have the characteristic roots of Equation (13) as

(15) $$\varrho_{1}=\frac{-\rho_{\text{V}}+\sqrt{\rho_{\text{V}}^{2}-4\rho_{\text{P}}}}{2},\varrho_{2}=\frac{-\rho_{\text{V}}-\sqrt{\rho_{\text{V}}^{2}-4\rho_{\text{P}}}}{2}.$$

(i) When $\rho_{\text{V}}^{2}>4\rho_{\text{P}}$, from Equation (15), we have $\rho_{\text{V}}>\sqrt{\left(\rho_{\text{V}}^{2}-4\rho_{\text{P}}\right)}>0$, thus real characteristic roots ϱ_1_ < 0 and ϱ_2_ < 0. Based on modern control theory (Tewari, 2002), there exists a nonsingular matrix *T* satisfying

(16) $$\Phi (t)=T\left[\begin{array}{cc} e^{\varrho_{1}t} & 0\\ 0 & e^{\varrho_{2}t} \end{array}\right]T^{-1}.$$

Substituting (16) into (14), we obtain that

$$\|\chi (t){\|}_{2}=\|\Phi (t)\chi (0){\|}_{2}⩽\|\Phi (t){\|}_{F}\|\chi (0){\|}_{2}⩽\|T{\|}_{F}\|T^{-1}{\|}_{F}\sqrt{e^{2\varrho_{1}t}+e^{2\varrho_{2}t}}\|\chi (0){\|}_{2}$$

is globally exponentially convergent to zero since ||*T*||_*F*_ and $||T^{-1}|{|}_{F}$ are limited. Therefore, the first element of χ(*t*), i.e., position error *E*_*f*_(*t*), is globally exponentially convergent to zero.

(ii) When $\rho_{\text{V}}^{2}=4\rho_{\text{P}}$, from Equation (15) we have real equal characteristic roots ϱ_1_ = ϱ_2_ = ϱ_*e*_ = −*ρ*_V_/2 < 0. Based on modern control theory (Tewari, 2002), there exists a nonsingular matrix *T* satisfying

(17) $$\Phi (t)=T\left[\begin{array}{cc} e^{\varrho_{e}t} & te^{\varrho_{e}t}\\ 0 & e^{\varrho_{e}t} \end{array}\right]T^{-1}.$$

Substituting (17) into (14), we obtain that

$$\|\chi (t){\|}_{2}=\|\Phi (t)\chi (0){\|}_{2}⩽\|\Phi (t){\|}_{F}\|\chi (0){\|}_{2}⩽\|T{\|}_{F}\|T^{-1}{\|}_{F}\sqrt{t^{2}+2}e^{\varrho_{e}t}\|\chi (0){\|}_{2}$$

is globally exponentially convergent to zero. Therefore, the first element of χ(*t*), i.e., position error *E*_*f*_(*t*), is globally exponentially convergent to zero.

(iii) When $\rho_{\text{V}}^{2}<4\rho_{\text{P}}$, from Equation (15) we have two imaginary characteristic roots and set them as ϱ_1_ = *σ* + *jω* and ϱ_2_ = *σ* − *jω* with the real part σ < 0. Based on modern control theory (Tewari, 2002),

(18) Φ(t)=[cos ωtsin ωt−sin ωtcos ωt]eσt.

Substituting (18) into (14), we obtain that

$$\|\chi (t){\|}_{2}=\|\Phi (t)\chi (0){\|}_{2}⩽\|\Phi (t){\|}_{F}\|\chi (0){\|}_{2}=\sqrt{2}e^{\sigma t}\|\chi (0){\|}_{2}$$

is globally exponentially convergent to zero. Therefore, the first element of χ(*t*), i.e., position error *E*_*f*_(*t*), is globally exponentially convergent to zero.

In conclusion, it is proved that the position error *E*_*f*_(*t*) is globally convergent to zero with the kind of error feedback in Equation (11) where *ρ*_V_ and *ρ*_P_ are both set positive.

### 2.3. NDSO subscheme of left/right arm

In order to remedy the joint-angle drift problem, a neural-dynamic based synchronous-optimization subscheme (Sub-NDSO) of left/right arm (i.e., the following theorem) is proposed.

**Theorem 2**. *For a left or right arm of dual-redundant-manipulators, given a closed end-effector path, i.e., *r*_L/R_(*T*) = *r*_L/R_(0) where *T* denotes a task execution period, if Equations (19)–(23) are satisfied, the left or right arm of dual-redundant-manipulators achieves repetitive motion, and the joint-drift θ_L/R_(*t*)−θ_L/R_(0) would converge exponentially to zero. In addition, all the joint-angles, joint-velocities and joint-accelerations are constrained within their limits, i.e.,*

(19) $$minimize\;\frac{1}{2}\|{\ddot{\theta }}_{L/R}(t)+b_{L/R}(t){\|}_{2}^{2}$$

(20) $$subject\;to\;J_{L/R}(\theta_{L/R}){\ddot{\theta }}_{L/R}(t)={\ddot{r}}_{afL/R}(t)$$

(21) $$\theta_{L/R}^{-}⩽\theta_{L/R}(t)⩽\theta_{L/R}^{+}$$

(22) $${\dot{\theta }}_{L/R}^{-}⩽{\dot{\theta }}_{L/R}(t)⩽{\dot{\theta }}_{L/R}^{+}$$

(23) $${\ddot{\theta }}_{L/R}^{-}⩽{\ddot{\theta }}_{L/R}(t)⩽{\ddot{\theta }}_{L/R}^{+}$$

$$\begin{array}{l} with\;b_{L/R}(t)=(\alpha +\beta ){\dot{\theta }}_{L/R}(t)+\alpha \beta (\theta_{L/R}(t)-\theta_{L/R}(0)),\\ \;{\ddot{r}}_{afL/R}(t)={\ddot{r}}_{\text{dL/R}}(t)-{\dot{J}}_{L/R}(\theta_{L/R}){\dot{\theta }}_{L/R}(t)\\ \;+\rho_{v}({\dot{r}}_{\text{dL/R}}(t)-J_{L/R}(\theta_{L/R}){\dot{\theta }}_{L/R}(t))+\rho_{p}(r_{\text{dL/R}}(t)\\ \;-f(\theta_{L/R})) \end{array}$$

*where ||·||_2_ denotes the two-norm of a vector; θ_L/R_, ${\dot{\theta }}_{\text{L/R}}$, and ${\ddot{\theta }}_{\text{L/R}}$ denote the joint angle, joint velocity, and joint acceleration of the left or right arms of dual-redundant-manipulators; *r*_dL/R_, ṙ_dL/R_, and ${\ddot{r}}_{\text{dL/R}}$ denote desired end-effector position, desired end-effector velocity, and desired end-effector acceleration of the left or right arm of dual-redundant-manipulators; *J*(θ) and $\dot{J}\left(\theta \right)$ are the Jacobian matrix and the first order derivative of Jacobian matrix; α > 0 and β > 0 are used to scale the joint displacement; $\theta_{\text{L/R}}^{\pm }$, ${\dot{\theta }}_{\text{L/R}}^{\pm }$ and ${\ddot{\theta }}_{\text{L/R}}^{\pm }$ denote the upper and lower limits of the joint angles, joint velocities and joint accelerations of the left/right manipulator, respectively*.

***Proof 2***. First, a vector-valued error function, i.e., the deviation between the joint instant angle θ_L/R_(*t*) and the initial joint angle θ_L/R_(0) of the left/right manipulator, is defined as

(24) $$e_{\text{1L/R}}(t)=\theta_{L/R}(t)-\theta_{L/R}(0).$$

The joint-angle drift is zero if and only if the value of the error function *e*_1L/R_(*t*) = 0. In order to reduce and eventually eliminate the joint displacement, by the neural-dynamic method, we can obtain

(25) $${\dot{e}}_{\text{1L/R}}(t)=-\alpha e_{\text{1L/R}}(t)=-\alpha [\theta_{L/R}(t)-\theta_{L/R}(0)],$$

where design parameter α is used to adjust the convergence rate of *e*_1L/R_(*t*) to zero. By taking the derivative of Equation (24) with time *t*, ${ė}_{\text{1L/R}}\left(t\right)={\dot{\theta }}_{\text{L/R}}\left(t\right)$ is obtained. Substituting it into Equation (25), the following equation is obtained, i.e.,

(26) $${\dot{\theta }}_{L/R}(t)+\alpha (\theta_{L/R}(t)-\theta_{L/R}(0))=0.$$

Second, in order to obtain the acceleration-level repetitive motion criterion, the joint acceleration should be included in the criterion. That is to say, there should be an equation equivalent to (26), which includes joint acceleration. To do so, the neural dynamic method is applied to Equation (26) again. Similarly, a vector-valued joint-displacement function is defined as

(27) $$e_{\text{2L/R}}(t)={\dot{\theta }}_{L/R}(t)+\alpha (\theta_{L/R}(t)-\theta_{L/R}(0)).$$

According to neural dynamic design method (Cai and Zhang, 2012), i.e.,

(28) $${\dot{e}}_{\text{2L/R}}(t)=-\beta e_{\text{2L/R}}(t)$$

where design parameter β > 0, we can get

(29) $${\ddot{\theta }}_{L/R}(t)+\alpha {\dot{\theta }}_{L/R}(t)=-\beta ({\dot{\theta }}_{L/R}(t)+\alpha (\theta_{L/R}(t)-\theta_{L/R}(0))).$$

Equation (29) is rewritten as

(30) $${\ddot{\theta }}_{L/R}(t)+(\alpha +\beta ){\dot{\theta }}_{L/R}(t)+\alpha \beta (\theta_{L/R}(t)-\theta_{L/R}(0))=0.$$

Considering the motion of the robot manipulator, it is better to minimize the performance $||{\ddot{\theta }}_{\text{L/R}}\left(t\right)+\left(\alpha +\beta \right){\dot{\theta }}_{\text{L/R}}\left(t\right)+\alpha \beta \left(\theta_{\text{L/R}}\left(t\right)-\theta_{\text{L/R}}\left(0\right)\right)|{|}_{2}^{2}/2$ rather than use (30) directly, i.e.,

(31) $$\text{minimize }\frac{1}{2}\|{\ddot{\theta }}_{L/R}(t)+b_{L/R}(t){\|}_{2}^{2},$$

where $b_{\text{L/R}}\left(t\right)=\left(\alpha +\beta \right){\dot{\theta }}_{\text{L/R}}\left(t\right)+\alpha \beta \left(\theta_{\text{L/R}}\left(t\right)-\theta_{\text{L/R}}\left(0\right)\right)$, and ||·||_2_ denotes the two-norm of a vector. If Equation (31) is used as the optimization criterion, the joint angle θ_L/R_(*t*) tends to converge to θ_L/R_(0). At the end of the task execution period, θ_L/R_(*T*) = θ_L/R_(0). Equation (19) is thus proved.

In practical applications, the joint physical limits, i.e., joint-angle limits, joint-velocity limits and joint-acceleration limits, should be considered into the scheme, and thus an NDSO subschemes (termed as Sub-NDSO) is obtained as

(32) $$\text{minimize }\frac{1}{2}\|{\ddot{\theta }}_{L/R}(t)+b_{L/R}(t){\|}_{2}^{2}$$

(33) $$\text{subject to }J_{L/R}(\theta_{L/R}){\ddot{\theta }}_{L/R}(t)={\ddot{r}}_{\text{aL/R}}(t)$$

(34) $$\theta_{L/R}^{-}⩽\theta_{L/R}(t)⩽\theta_{L/R}^{+}$$

(35) $${\dot{\theta }}_{L/R}^{-}⩽{\dot{\theta }}_{L/R}(t)⩽{\dot{\theta }}_{L/R}^{+}$$

(36) $${\ddot{\theta }}_{L/R}^{-}⩽{\ddot{\theta }}_{L/R}(t)⩽{\ddot{\theta }}_{L/R}^{+}$$

$$\begin{array}{l} \text{with }b_{L/R}(t)=(\alpha +\beta ){\dot{\theta }}_{L/R}(t)+\alpha \beta (\theta_{L/R}(t)-\theta_{L/R}(0))\\ \;\;\;{\ddot{r}}_{\text{aL/R}}(t)={\ddot{r}}_{\text{dL/R}}(t)-{\dot{J}}_{L/R}(\theta_{L/R}){\dot{\theta }}_{L/R}(t) \end{array}$$

where α > 0 and β > 0 are used to scale the joint displacement.

According to the acceleration-level feedback error design method in Theorem 1, ${\ddot{r}}_{\text{aL/R}}$ in Equation (33) can be replaced by ${\ddot{r}}_{\text{afL/R}}\left(t\right)={\ddot{r}}_{\text{dL/R}}\left(t\right)-{\dot{J}}_{\text{L/R}}\left(\theta_{\text{L/R}}\right){\dot{\theta }}_{\text{L/R}}\left(t\right)+\rho_{\text{v}}\left({ṙ}_{\text{dL/R}}\left(t\right)-J_{\text{L/R}}\left(\theta_{\text{L/R}}\right){\dot{\theta }}_{\text{L/R}}\left(t\right)\right)+\rho_{\text{p}}\left(r_{\text{dL/R}}\left(t\right)-f\left(\theta_{\text{L/R}}\right)\right)$. Equations (19)–(23) is thus proved. That is to say, with Equations (19)–(23), the left or right arm of dual-redundant-manipulators can achieve repetitive motion, meanwhile it can avoid joint-physical limits during the execution of the task.

Next, the exponential convergence rate of joint-drift θ_L/R_(*t*)−θ_L/R_(0) will be proved. In the light of differential equation theory (Hartman and Philip, 1982), the *i*th element of *e*_2L/R_(*t*) in Equation (28) is

(37) $$e_{\text{2L/Ri}}(t)=e_{\text{2L/Ri}}(0)e^{-\beta t}.$$

When *t* approaches to infinity, each element would approach exponentially zero, i.e.,

(38) $$\underset{t\to \infty }{\lim}e_{\text{2L/Ri}}(t)=\underset{t\to \infty }{\lim}e_{\text{2L/Ri}}(0)e^{-\beta t}=0.$$

The proof of Theorem 2 is completed.

### 2.4. NDSO scheme

In this section, based on the neural-dynamic based synchronous-optimization subschemes (Sub-NDSO) of the left arm and right arm proposed in Theorem 2, a neural-dynamic based synchronous-optimization scheme of dual redundant robot manipulators (NDSO) is proposed and developed.

**Theorem 3**. *For a dual-redundant-manipulators system, including left manipulator and right manipulator, given a closed end-effector path, i.e., *r*(*T*) = *r*(0) where *T* denotes a task execution period, if Equations (39)–(43) are satisfied, the dual-redundant-manipulators will achieve repetitive motion, and the joint-drift θ(*t*)−θ(0) would converge exponentially to zero. In addition, all the joint angles, joint velocities and joint accelerations of the dual-redundant-manipulators are constrained within their limits, i.e.*,

(39) $$minimize\;\frac{1}{2}{\ddot{\theta }}^{\text{T}}(t)\ddot{\theta }(t)+b^{\text{T}}(t)\ddot{\theta }(t)$$

(40) $$subject\;to\;J(\theta )\ddot{\theta }(t)={\ddot{r}}_{af}(t)$$

(41) $$\theta^{-}⩽\theta (t)⩽\theta^{+}$$

(42) $${\dot{\theta }}^{-}⩽\dot{\theta }(t)⩽{\dot{\theta }}^{+}$$

(43) $${\ddot{\theta }}^{-}⩽\ddot{\theta }(t)⩽{\ddot{\theta }}^{+}$$

$$\begin{array}{l} with\;b(t)=(\alpha +\beta )\dot{\theta }(t)+\alpha \beta (\theta (t)-\theta (0)),\\ \;\;\;{\ddot{r}}_{af}(t)={\ddot{r}}_{d}(t)-\dot{J}(\theta )\dot{\theta }(t)+\rho_{v}({\dot{r}}_{d}(t)-J(\theta )\dot{\theta }(t))\\ \;\;\;+\rho_{p}(r_{d}(t)-f(\theta )) \end{array}$$

where $\theta \left(t\right)={\left[\theta_{\text{L}}\left(t\right),\theta_{\text{R}}\left(t\right)\right]}^{\text{T}}$, $\dot{\theta }\left(t\right)={\left[{\dot{\theta }}_{\text{L}}\left(t\right),{\dot{\theta }}_{\text{R}}\left(t\right)\right]}^{\text{T}}$, and $\ddot{\theta }\left(t\right)={\left[{\ddot{\theta }}_{\text{L}}\left(t\right),{\ddot{\theta }}_{\text{R}}\left(t\right)\right]}^{\text{T}}$ denote the joint angle, joint velocity, and joint acceleration of the dual-redundant-manipulators; $r_{\text{d}}\left(t\right)={\left[r_{\text{dL}}\left(t\right),r_{\text{dR}}\left(t\right)\right]}^{\text{T}}$, ${ṙ}_{\text{d}}\left(t\right)={\left[{ṙ}_{\text{dL}}\left(t\right),{ṙ}_{\text{dR}}\left(t\right)\right]}^{\text{T}}$, and ${\ddot{r}}_{\text{d}}\left(t\right)={\left[{\ddot{r}}_{\text{dL}}\left(t\right),{\ddot{r}}_{\text{dR}}\left(t\right)\right]}^{\text{T}}$ denote the position vector, velocity vector, and acceleration vector of the end-effector of the dual-redundant-manipulators; Scalar parameters α > 0 and β > 0 are used to scale the joint displacements; $\theta^{\pm }={\left[\theta_{L}^{\pm },\theta_{R}^{\pm }\right]}^{T}$, ${\dot{\theta }}^{\pm }={\left[{\dot{\theta }}_{L}^{\pm },{\dot{\theta }}_{R}^{\pm }\right]}^{T}$ and ${\ddot{\theta }}^{\pm }={\left[{\ddot{\theta }}_{L}^{\pm },{\ddot{\theta }}_{R}^{\pm }\right]}^{T}$ denote the upper and lower limits of the joint angles, joint velocities and joint accelerations of the dual-redundant-manipulator, respectively. The combined Jacobian matrix and the first order derivative of the combined Jacobian matrix of the dual-redundant-manipulators are

(44) $$J(\theta )=\left[\begin{array}{c} J_{L}(\theta_{L})\;\;\;0\\ \;\;0\;\;\;\;\;J_{R}(\theta_{R}) \end{array}\right],\;\dot{J}(\theta )=\left[\begin{array}{c} {\dot{J}}_{L}(\theta_{L})\;\;\;0\\ \;\;0\;\;\;\;\;{\dot{J}}_{R}(\theta_{R}) \end{array}\right].$$

***Proof 3***. Firstly, the optimization criterion (32) can be simplified as

(45) $$\begin{array}{l} \;\frac{1}{2}{\left\|{\ddot{\theta }}_{\text{L}/\text{R}}(t)+b_{\text{L/R}}(t)\right\|}_{2}^{2}\\ =\frac{1}{2}{\left({\ddot{\theta }}_{\text{L}/\text{R}}(t)+b_{\text{L}/\text{R}}(t)\right)}^{\text{T}}\left({\ddot{\theta }}_{\text{L}/\text{R}}(t)+b_{\text{L}/\text{R}}(t)\right)\\ =\frac{1}{2}({\ddot{\theta }}_{\text{L}/\text{R}}^{\text{T}}(t){\ddot{\theta }}_{\text{L}/\text{R}}(t)+{\ddot{\theta }}_{\text{L}/\text{R}}^{\text{T}}(t)b_{\text{L}/\text{R}}(t)+b_{\text{L}/\text{R}}^{\text{T}}(t){\ddot{\theta }}_{\text{L}/\text{R}}(t)\\ \;+b_{\text{L}/\text{R}}^{\text{T}}(t)b_{\text{L}/\text{R}}(t))\\ =\frac{1}{2}{\ddot{\theta }}_{\text{L}/\text{R}}^{\text{T}}(t){\ddot{\theta }}_{\text{L}/\text{R}}(t)+b_{\text{L}/\text{R}}^{\text{T}}(t){\ddot{\theta }}_{\text{L}/\text{R}}(t)+\frac{1}{2}b_{\text{L}/\text{R}}^{\text{T}}(t)b_{\text{L}/\text{R}}(t). \end{array}$$

Since the redundant resolution problem is solved at the joint-acceleration level and ${\ddot{\theta }}_{\text{L/R}}$ is the decision variable, $b_{\text{L/R}}^{\text{T}}\left(t\right)b_{\text{L/R}}\left(t\right)/2$ in Equation (45) can be regarded as a constant. Therefore, minimizing $||{\ddot{\theta }}_{\text{L/R}}\left(t\right)+b_{\text{L/R}}\left(t\right)|{|}_{2}^{2}/2={\ddot{\theta }}_{\text{L/R}}^{\text{T}}\left(t\right){\ddot{\theta }}_{\text{L/R}}\left(t\right)/2+b_{\text{L/R}}^{\text{T}}\left(t\right){\ddot{\theta }}_{\text{L/R}}\left(t\right)+b_{\text{L/R}}^{\text{T}}\left(t\right)b_{\text{L/R}}\left(t\right)/2$is equivalent to minimizing ${\ddot{\theta }}_{\text{L/R}}^{\text{T}}\left(t\right){\ddot{\theta }}_{\text{L/R}}\left(t\right)/2+b_{\text{L/R}}^{\text{T}}\left(t\right){\ddot{\theta }}_{\text{L/R}}\left(t\right)$. Combining the joint variables of left and right manipulators into one combined vector, the optimization criterion can be written as

(46) $$\text{minimize }{\ddot{\theta }}^{\text{T}}(t)\ddot{\theta }(t)/2+b^{\text{T}}(t)\ddot{\theta }(t)$$

where $\ddot{\theta }\left(t\right)={\left[{\ddot{\theta }}_{\text{L}}\left(t\right),{\ddot{\theta }}_{\text{R}}\left(t\right)\right]}^{\text{T}}$ and $b\left(t\right)={\left[b_{\text{L}}\left(t\right),b_{\text{R}}\left(t\right)\right]}^{\text{T}}$.

Secondly, acceleration level forward kinematic Equation (20) of left and right manipulators can be written as a combined forward kinematic equation as

(47) $$\left[\begin{array}{c} J_{\text{L}}(\theta )\;\;\;0\\ \;\;0\;\;\;\;\;J_{\text{R}}(\theta ) \end{array}\right]\cdot \left[\begin{array}{c} {\ddot{\theta }}_{\text{L}}(t)\\ {\ddot{\theta }}_{\text{R}}(t) \end{array}\right]=\left[\begin{array}{c} {\ddot{r}}_{\text{afL}}(t)\\ {\ddot{r}}_{\text{afR}}(t) \end{array}\right]$$

where

(48) $$\begin{array}{l} {\ddot{r}}_{\text{afL}}(t)={\ddot{r}}_{\text{dL}}(t)-{\dot{J}}_{\text{L}}(\theta_{\text{L}}){\dot{\theta }}_{\text{L}}(t)\\ \;+\rho_{\text{v}}({\dot{r}}_{\text{dL}}(t)-J_{\text{L}}(\theta_{\text{L}}){\dot{\theta }}_{\text{L}}(t))+\rho_{\text{p}}(r_{\text{dL}}(t)-f(\theta_{\text{L}})), \end{array}$$

(49) $$\begin{array}{l} {\ddot{r}}_{\text{afR}}(t)={\ddot{r}}_{\text{dR}}(t)-{\dot{J}}_{\text{R}}(\theta_{\text{R}}){\dot{\theta }}_{\text{R}}(t)\\ \;+\rho_{\text{v}}({\dot{r}}_{\text{dR}}(t)-J_{\text{R}}(\theta_{\text{R}}){\dot{\theta }}_{\text{R}}(t))+\rho_{\text{p}}(r_{\text{dR}}(t)-f(\theta_{\text{R}})). \end{array}$$

Combining the upper and lower joint-limits of left and right arms of dual-redundant-manipulators, we can get combined joint-angular, joint-velocity, joint-acceleration limits respectively as

(50) $$\theta^{\pm }\left(t\right)={\left[\theta_{\text{L}}^{\pm }\left(t\right),\theta_{\text{R}}^{\pm }\left(t\right)\right]}^{\text{T}},$$

(51) $${\dot{\theta }}^{\pm }\left(t\right)={\left[{\dot{\theta }}_{\text{L}}^{\pm }\left(t\right),{\dot{\theta }}_{\text{R}}^{\pm }\left(t\right)\right]}^{\text{T}},$$

(52) $${\ddot{\theta }}^{\pm }\left(t\right)={\left[{\ddot{\theta }}_{\text{L}}^{\pm }\left(t\right),{\ddot{\theta }}_{\text{R}}^{\pm }\left(t\right)\right]}^{\text{T}}.$$

Taking into consideration of optimization criterion (46), feedback considered acceleration-level kinematic equation (47), and joint-limits (50)–(52), NDSO scheme (40)–(43) is obtained. The proof of Theorem 3 is completed.

## 3. Quadratic programming unification & solver

In this section, the proposed NDSO scheme (39)–(43) is unified into a standard quadratic programming problem, which is equivalent to linear variational inequality problem and is further equivalent to a piecewise linear projection equation. Finally, the piecewise linear projection equation is solved by a linear variational inequalities-based primal-dual neural network (LVI-PDNN).

### 3.1. Joint-limits conversion

In order to resolve the redundancy problem at the acceleration-level and satisfy the format requirement of standard quadratic programming, physical limits (41)–(43) at different levels should be converted into one bound constraint with joint-acceleration $\ddot{\theta }\left(t\right)$. Specifically, the *i*th elements of bounds ζ^−^ and ζ^+^ are defined respectively as

$$\begin{array}{l} \zeta_{i}^{-}(t)=\text{max}\{{\ddot{\theta }}_{i}^{-}(t),\lambda_{\text{v}}({\dot{\theta }}_{i}^{-}-{\dot{\theta }}_{i}(t)),\lambda_{\text{p}}((\theta_{i}^{-}+\vartheta_{i})-\theta_{i}(t))\},\\ \zeta_{i}^{+}(t)=\text{min}\{{\ddot{\theta }}_{i}^{+}(t),\lambda_{\text{v}}({\dot{\theta }}_{i}^{+}-{\dot{\theta }}_{i}(t)),\lambda_{\text{p}}((\theta_{i}^{+}-\vartheta_{i})-\theta_{i}(t))\}. \end{array}$$

Actually, there exist the inertia movement during the deceleration period caused by the mechanical inertia of the dual-redundant-manipulators in practice. Thus critical areas for joint position variables are considered into physical limits' representation so that there will appear a deceleration earlier when they enter the areas but not reach the joint position limit yet. *ϑ*_*i*_ > 0 is a small constant and used to define the critical areas $\left[\theta_{i}^{-},\theta_{i}^{-}+\vartheta_{i}\right]$ and $\left[\theta_{i}^{+}-\vartheta_{i},\theta_{i}^{+}\right]$. In the simulation section of the paper, *ϑ*_*i*_ > 0 is set 0.01. The coefficient λ_v_ > 0 and λ_p_ > 0 denote the decreasing amplitude (Zhang et al., 2008).

Therefore, constraints (39)–(43) can be rewritten as

(53) $$\text{minimize }\frac{1}{2}\ddot{\theta }{\left(t\right)}^{\text{T}}W\ddot{\theta }(t)+b^{\text{T}}(t)\ddot{\theta }(t)$$

(54) $$\begin{array}{l} \text{subject to }J\left(\theta \right)\ddot{\theta }\left(t\right)={\ddot{r}}_{\text{af}}\left(t\right) \end{array}$$

(55) $$\;\zeta^{-}\left(t\right)⩽\ddot{\theta }\left(t\right)⩽\zeta^{+}\left(t\right)$$

The scheme (53)–(55) can be further unified into the following standard quadratic programming

(56) $$\text{minimize }\frac{1}{2}x^{\text{T}}Gx+d^{\text{T}}x$$

(57) subject to  Cx=h

(58) $$\;x^{-}⩽x⩽x^{+}$$

where

$$\begin{array}{l} x=\ddot{\theta }(t)=\left[\begin{array}{c} {\ddot{\theta }}_{\text{L}}(t)\\ {\ddot{\theta }}_{\text{R}}(t) \end{array}\right]\in R^{2n},\;G=W=\left[\begin{array}{cc} 1 & 0\\ 0 & 1 \end{array}\right]\in R^{2n\times 2n},\\ d=b(t)=\left[\begin{array}{c} b_{\text{L}}(t)\\ b_{\text{R}}(t) \end{array}\right]\in R^{2n},\;h={\ddot{r}}_{\text{af}}(t)=\left[\begin{array}{c} {\ddot{r}}_{\text{afL}}(t)\\ {\ddot{r}}_{\text{afR}}(t) \end{array}\right]\in R^{2m},\\ C=J=\left[\begin{array}{cc} J_{\text{L}}(\theta_{\text{L}}) & 0\\ 0 & J_{\text{R}}(\theta_{\text{R}}) \end{array}\right]\in R^{2m\times 2n},\;x^{\pm }=\zeta^{\pm }(t)\in R^{2n}. \end{array}$$

### 3.2. Quadratic programming solver

According to Zhang et al. (2008), finding the solutions to quadratic programming problem (56)–(58) is equivalent to finding out a primal-dual equilibrium vector u^*^ = [x^*^; η^*^]^T^ ∈ Ω: = {*u* = [*x*^T^, η^T^]^T^ ∈ *R*^2*n*+2*m*^|*u*^−^ ≤ *u* ≤ *u*^+^} to the following linear variational inequality

(59) $${\left(u-u^{*}\right)}^{\text{T}}(Mu^{*}+q)⩾0,\forall u\in \Omega ,$$

where the augmented primal-dual decision variable *u* ∈ *R*^(2*n*+2*m*)^, and its bounds *u*^±^ ∈ *R*^(2*n*+2*m*)^ are respectively defined as

$$u=\left[\begin{array}{c} x\\ \eta \end{array}\right],\;u^{+}=\left[\begin{array}{c} x^{+}\\ 1_{\nu }\varpi \end{array}\right],\;u^{-}=\left[\begin{array}{c} x^{-}\\ -1_{\nu }\varpi \end{array}\right],$$

with η ∈ *R*^2*m*^ being the corresponding dual decision vectors of Equation (57), $1_{\nu }={\left[1,\cdots \;,1\right]}^{\text{T}}$ denoting an appropriately-dimensioned vector composed of ones, and *ϖ* = 10^10^ ∈ *R* replacing the +∞ for simulation and implementation purposes. The matrix *M* ∈ *R*^(2*n*+2*m*) × (2*n*+2*m*)^ and the vector *q* ∈ *R*^2*n*+2*m*^ are defined respectively as

$$M=\left[\begin{array}{cc} G & -C^{\text{T}}\\ C & 0 \end{array}\right],q=\left[\begin{array}{c} d\\ -h \end{array}\right].$$

The above inequality problem (59) can be solved by the following piecewise-linear projection equation (Zhang and Zhang, 2013a) as

(60) $$P_{\Omega }(u-(Mu+q))-u=0$$

where $P_{\Omega }\left(\cdot \right)\in R^{2n+2m}\to \Omega \subset R^{2n+2m}$ is a projection operator defined from *R*^2*n*+2*m*^ onto Ω, and the *i*th element of *P*_Ω_(*u*) is

$$\left\{ \begin{array}{l} u_{\text{i}}^{-},\;if\;u_{\text{i}}<u_{\text{i}}^{-}\\ u_{\text{i}},\;if\;u_{\text{i}}^{-}<u_{\text{i}}<u_{\text{i}}^{+},\forall i\in \{1,2,\cdots ,2n+2m\}\\ u_{\text{i}}^{+},\;if\;u_{\text{i}}>u_{\text{i}}^{+} \end{array}\right.$$

According to previous design experience on recurrent neural networks (Zhang and Zhang, 2013a), a linear-variational-inequality-based primal-dual neural network (abbreviated as LVI-PDNN) is employed to solve the piecewise-linear projection Equation (60) as well as the quadratic programming problem (56)–(58), i.e.,

(61) $$\dot{u}=\gamma (I+M^{\text{T}})(P_{\Omega }(u-(Mu+q))-u),$$

where *I* is an identity matrix, and parameter γ ∈ *R* is a positive design parameter designed to scale the convergence rate of neural network. From Zhang and Zhang (2013a), the state vector *u*(*t*) of the primal-dual neural network in Equation (61) is globally convergent to an equilibrium point u^*^. Furthermore, the first 2*n* elements of u^*^ constitute the solutions to the original quadratic programming problem (56)–(58).

Considering the systematic error generally including the differentiation error and the implementation error, the perturbed LVI-PDNN is formulated as

(62) $$\dot{u}=\gamma (I+M^{\text{T}}+\Delta D)(P_{\Omega }(u-(Mu+q))-u)+\Delta S,$$

where Δ*D* ∈ *R*^(2*n*+2*m*) × (2*n*+2*m*)^ and Δ*S* ∈ *R*^2*n*+2*m*^ denote the differentiation error matrix and the implementation error vector respectively. Equation (62) will be used in the experiment on robustness verification.

## 4. Computer simulations

In this section, the dual PA10 robot manipulators synthesized by the presented NDSO scheme are expected to track three closed complex trajectories, i.e., a pentagram, number “47” writing and end-effector-coupled pentagram. Each manipulator has 7 degrees-of-freedom, and the dual-manipulators have 14 degrees-of-freedom in total. All joint physical limits are shown in Table 1. The design parameter α and β are set 4, and the design parameter γ = 10^5^ in the ensuing simulations.

**Table 1:** Joint physical limits used in simulations.

**#**	**$\theta_{\text{L}}^{-}$**	**$\theta_{\text{L}}^{+}$**	**$\theta_{\text{R}}^{-}$**	**$\theta_{\text{R}}^{+}$**	**${\dot{\theta }}_{\text{L/R}}^{-}$**	**${\dot{\theta }}_{\text{L/R}}^{+}$**	**${\ddot{\theta }}_{\text{L/R}}^{-}$**	**${\ddot{\theta }}_{\text{L/R}}^{+}$**
** **	**(rad)**	**(rad)**	**(rad)**	**(rad)**	**(rad/s)**	**(rad/s)**	**(rad/s^2^)**	**(rad/s^2^)**
1	–1	1	–5	0	–1.5	1.5	–6	6
2	–2	2	–2	0	–1.5	1.5	–6	6
3	–1	1	–1	1	–1.5	1.5	–6	6
4	1	3	1	3	–1.5	1.5	–6	6
5	–1	1	–1	1	–1.5	1.5	–6	6
6	–2	0	–2	0	–1.5	1.5	–6	6
7	–1	1	–1	1	–1.5	1.5	–6	6

### 4.1. Pentagram path-tracking

**Figure 3:**
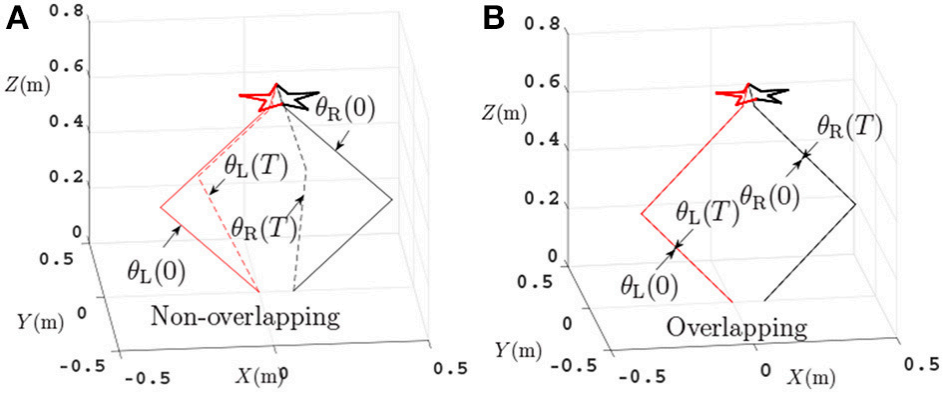
Comparisons between the scheme without considering repetitive motion and the NDSO scheme when tracking a pentagram-path. **(A)** Final states do not coincide with the initial states when using the scheme without considering repetitive motion. **(B)** Final states coincide with initial states when using NDSO scheme considering repetitive motion.

In this section, the dual PA10 robot manipulators are expected to cooperatively track a pentagram-path. Initial joint angles of the left arm are θ_L_(0) = [0; −π/4;0; π/2; 0; −π/6; 0] rad, and initial joint angles of the right arm are θ_R_(0) = [−π; −π/4; 0; π/2; 0; −π/6; 0] rad. The task execution period is 4 s. For comparisons, four sets of equations in which the variables $d,x^{-},x^{+},\rho_{\text{p}},\rho_{\text{v}}$ in Equation (56)–(58) are set different values are showed in Table 2. Then the four sets of equations make up three groups of contrast experiments which are performed to prove the efficiency of repetitive motion criterion, physical limits criterion and feedback criterion. Firstly, comparison results between the scheme considering physical-limits, feedback criteria but no repetitive motion criterion (experiment 1) and the NDSO scheme considering the repetitive motion, physical limits and feedback criteria (experiment 4) performed on dual PA10 robot manipulators are illustrated in Figures 3A,B, respectively. Figure 3A shows that the final states of the end-effectors of the left and right arms of the dual-redundant-manipulators do not coincide with the initial states, which means that the end-effectors of the dual-redundant-manipulators can not return to the initial states when the task is completed. That is to say, the joint drift phenomenon has happened. It is noticed that this phenomenon is not expected in practical applications because it is necessary to add extra self-motion to readjust the manipulator's configuration at the end of each task execution period in the cyclic motions. Evidently, this approach is inefficient. To remedy this joint-drift problem, the repetitive motion planning criterion is developed, and the corresponding result is shown in Figure 3B. Evidently, the final states of the dual-redundant-manipulators coincide well with their initial states. Comparing Figures 3A,B, we can see that the NDSO scheme nearly eliminates the joint-drift phenomena since it considers the repetitive motion criterion, and the efficiency of repetitive motion criterion is verified.

**Table 2:** Four sets of equations used in the three groups of contrast experiments.

	**1**	**2**	**3**	**4**
*d*	0∈*R*^2*n*^	*b*(*t*) ∈ *R*^2*n*^	*b*(*t*) ∈ *R*^2*n*^	*b*(*t*) ∈ *R*^2*n*^
*x*^−^	ζ^−^	−ϖζ^−^	ζ^−^	ζ^−^
*x*^+^	ζ^+^	ϖζ^+^	ζ^+^	ζ^+^
ρ_p_	1	1	0	1
ρ_v_	200	200	0	200

**Figure 4:**
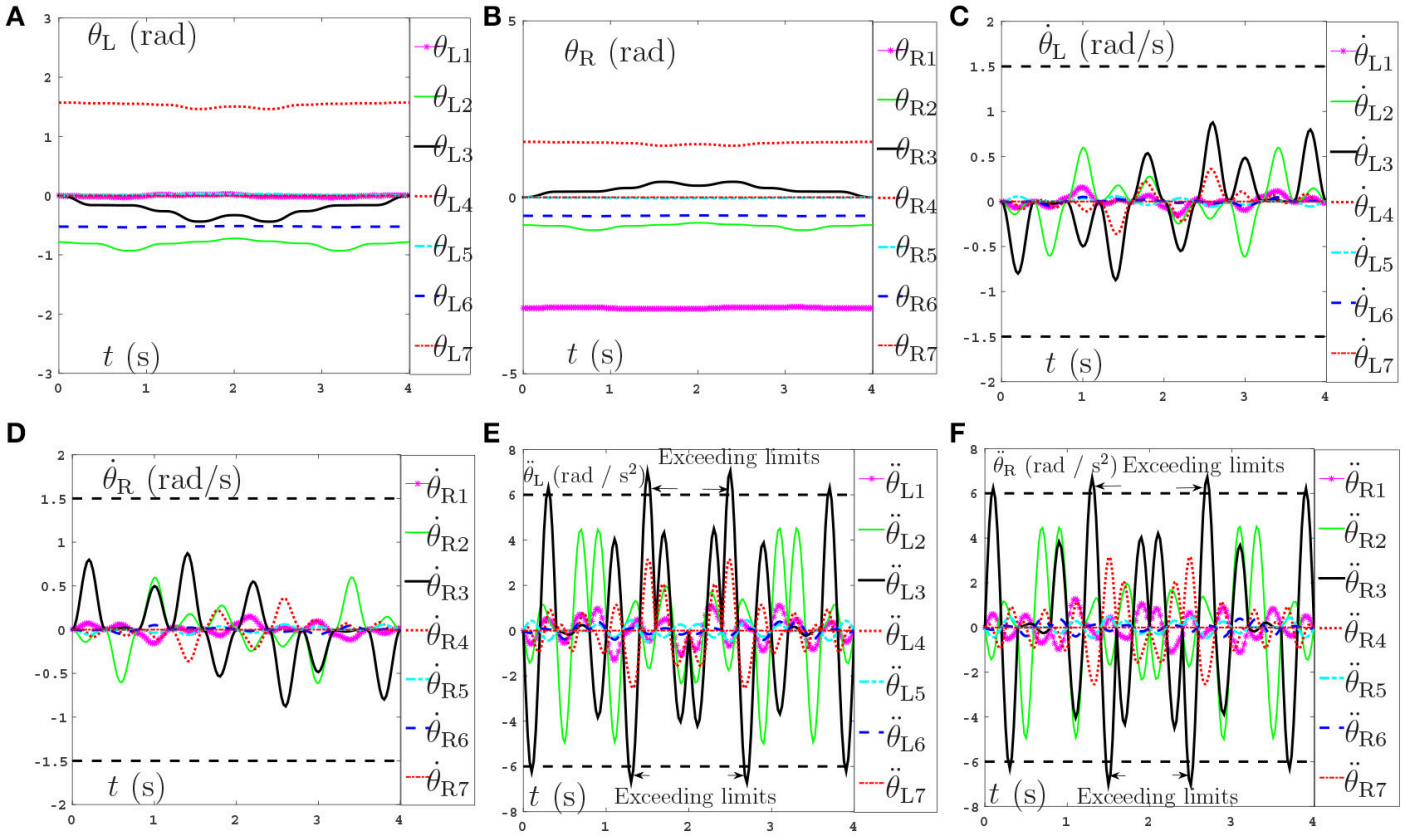
Joint angles, joint velocities, joint accelerations during the dual-redundant manipulators tracking a pentagram path when using the scheme with considering repetitive motion planning and feedback criteria but no physical-limits criterion. **(A)** Joint angle of left arm θ_L_. **(B)** Joint angle of right arm θ_R_. **(C)** Joint velocity of left arm ${\dot{\theta }}_{\text{L}}$. **(D)** Joint velocity of right arm ${\dot{\theta }}_{\text{R}}$. **(E)** Joint acceleration of left arm ${\ddot{\theta }}_{\text{L}}$. **(F)** Joint acceleration of right arm ${\ddot{\theta }}_{\text{R}}$.

**Figure 5:**
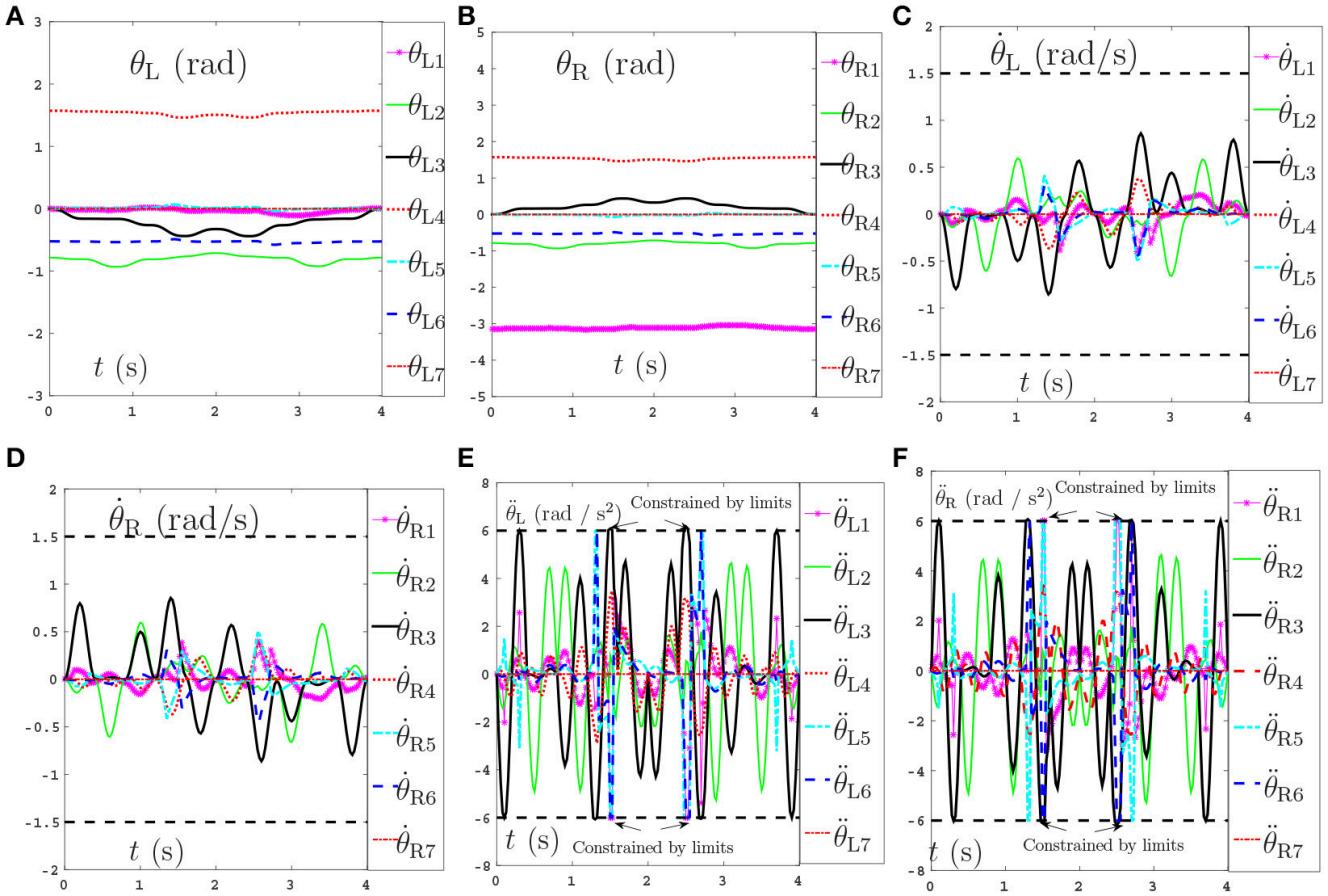
Joint angles, joint velocities, joint accelerations during the dual-redundant manipulators tracking a pentagram path when using the NDSO scheme with considering repetitive motion planning and physical-limits and feedback criterion. **(A)** Joint angle of left arm θ_L_. **(B)** Joint angle of right arm θ_R_. **(C)** Joint velocity of left arm ${\dot{\theta }}_{\text{L}}$. **(D)** Joint velocity of right arm ${\dot{\theta }}_{\text{R}}$. **(E)** Joint acceleration of left arm ${\ddot{\theta }}_{\text{L}}$. **(F)** Joint acceleration of right arm ${\ddot{\theta }}_{\text{R}}$.

Secondly, comparisons between the scheme with considering the repetitive motion planning and feedback criterion but without considering limits (experiment 2) and the NDSO scheme with considering the limits criterion (experiment 4) are illustrated in Figures 4, 5, respectively. The joint angles are shown in Figures 4C,D, 5C,D. We can see that the final states of joints coincide with the initial ones and thus the efficiency of the repetitive motion planning criterion are illustrated. The velocities are shown in Figures 4C,D, 5C,D. It can be seen from the figures that the velocities start from zero and end at zero, which is fit with the actual situations. However, Figures 4E,F show that ${\ddot{\theta }}_{\text{L3}}$ and ${\ddot{\theta }}_{\text{R3}}$ exceed their upper or lower acceleration limits in 0–4s. This may lead to the damage to the dual-redundant-manipulators and is less desirable for practical applications. By comparison, joint accelerations ${\ddot{\theta }}_{\text{L3}}$ and ${\ddot{\theta }}_{\text{R3}}$ in Figures 4E,F reach but never exceed their acceleration limits. This comparison result verifies the efficiency of the physical limits are very useful in applications.

**Figure 6:**
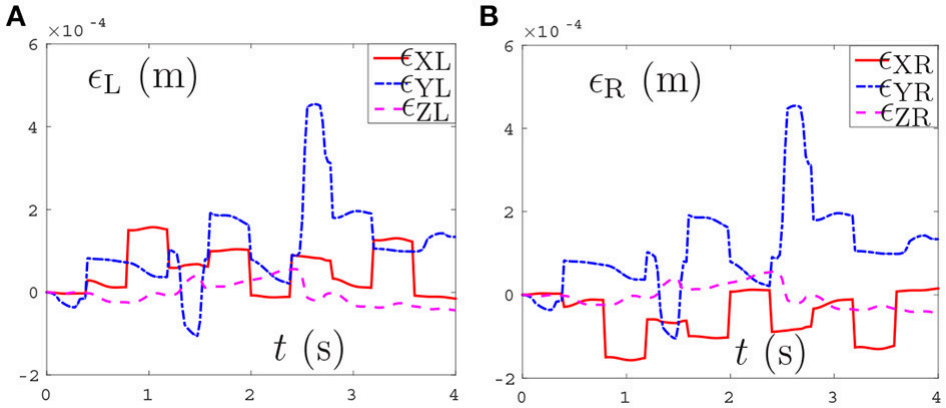
Position errors during the period of pentagram-path tracking synthesized by NDSO scheme without considering position and velocity feedback. **(A)** Position error of left arm ϵ_L_; **(B)** Position error of right arm ϵ_R_.

**Figure 7:**
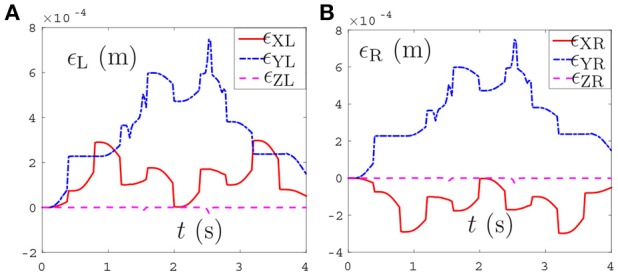
Position errors during the period of pentagram-path tracking synthesized by NDSO scheme with considering position and velocity feedback. **(A)** Position error of left arm ϵ_L_; **(B)** Position error of right arm ϵ_R_.

Thirdly, comparisons between the NDSO scheme without considering feedback (experiment 3) and the NDSO scheme proposed in this paper with considering feedback (experiment 4) are illustrated in Figures 6, 7, respectively. In the NDSO scheme, the feedback parameters ρ_P_ and ρ_V_ are set as 1 and 200, respectively. It can be seen from Figure 6 that the end-effector position errors of left and right arms are less than 6.0 × 10^−4^ m. However, the position errors become bigger and bigger as the task execution, i.e., the trend of the position errors are diverging. This would lead to bigger accumulated errors if the scheme is applied to perform cyclic tasks. Contrastively, the position errors in Figures 7A,B show that position errors are very tiny and become smaller and smaller since the proposed NDSO scheme is applied.

Last but not least, the joint drifts are measured when the position, velocity and acceleration feedback are taken into consideration in the NDSO scheme. Table 3 lists small joint drifts which are all less than 6.2 × 10^−3^ rads when the dual-redundant-manipulators track a pentagram-path synthesized by NDSO scheme.

**Table 3:** Joint drifts when dual-redundant-manipulators tracking a pentagram-path synthesized by NDSO scheme with considering repetitive motions, joint limits, and feedback.

	**Joint displacements (rad)**	**Joint displacements (degree)**
**LEFT ARM**		
θ_L1_(4)−θ_L1_(0)	+3.68924 × 10^−3^	+0.21138
θ_L2_(4)−θ_L2_(0)	+1.12535 × 10^−4^	+0.00645
θ_L3_(4)−θ_L3_(0)	−8.00902 × 10^−4^	−0.04589
θ_L4_(4)−θ_L4_(0)	+6.05971 × 10^−5^	+0.00347
θ_L5_(4)−θ_L5_(0)	−6.14706 × 10^−3^	−0.35220
θ_L6_(4)−θ_L6_(0)	−1.44414 × 10^−3^	−0.08274
θ_L7_(4)−θ_L7_(0)	0.00000	0.00000
**RIGHT ARM**		
θ_R1_(4)−θ_R1_(0)	−3.68924 × 10^−3^	−0.21138
θ_R2_(4)−θ_R2_(0)	+1.12535 × 10^−4^	+0.00645
θ_R3_(4)−θ_R3_(0)	+8.00902 × 10^−4^	+0.04589
θ_R4_(4)−θ_R4_(0)	+6.05971 × 10^−5^	+0.00347
θ_R5_(4)−θ_R5_(0)	+6.14706 × 10^−3^	+0.35220
θ_R6_(4)−θ_R6_(0)	−1.44414 × 10^−3^	−0.08274
θ_R7_(4)−θ_R7_(0)	0.00000	0.00000

In a word, the above three comparison experiments on tracking a pentagram-path illustrate well the effectiveness, safety and accuracy of the proposed NDSO scheme (39)–(43) and the LVI-PDNN to solve the joint-drift problem.

### 4.2. Number writing

In order to further verify the effectiveness, accuracy and generalization of the proposed NDSO scheme (39)–(43), another new end-effector task, i.e., number “47” writing, is expected to finished by the same dual PA10 robot manipulators which is synthesized by the NDSO scheme. In the simulations, ρ_P_ and ρ_V_ in Equations (48) and (49) are set as 1 and 100 respectively. Initial joint angles of the left arm are θ_L_(0) = [0; −π/4; 0; π/2; 0; −π/6; 0] rad, and initial joint angles of the right arm are θ_R_(0) = [−π; −π/4;0;π/2; 0; −π/6; 0] rad. The task execution period is 2s.

**Figure 8:**
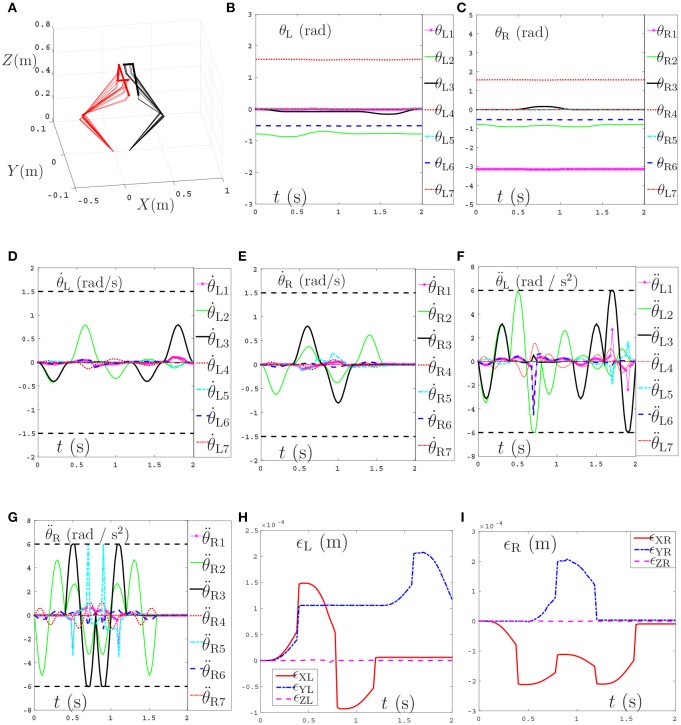
Tracking trajectories, joint angles, joint velocities, and joint accelerations during the period of number “47” writing synthesized by the proposed NDSO scheme (39)–(43) which considers repetitive motion planning, joint limits, and feedbacks. **(A)** 3-D simulation tracking trajectories. **(B)** Left arm joint angle θ_L_. **(C)** Right arm joint angle θ_R_. **(D)** Left arm joint velocity ${\dot{\theta }}_{\text{L}}$. **(E)** Right arm joint velocity ${\dot{\theta }}_{\text{R}}$. **(F)** Left arm joint acceleration ${\ddot{\theta }}_{\text{L}}$. **(G)** Right arm joint acceleration ${\ddot{\theta }}_{\text{R}}$. **(H)** Position error of left arm ϵ_L_. **(I)** Position error of right arm ϵ_R_.

The tracking trajectories, joint angles, joint velocities, joint accelerations and end-effector position errors are shown in Figure 8, and the joint drifts between the final state and the initial states of the left and right arms are listed in Table 4. As can be seen from Figure 8A, the end-effector task, i.e., number “47” writing is finished by the dual-redundant-manipulators synthesized by NDSO scheme (39)–(43) very well. In addition, as is shown in Figures 8B–E, all joint angles and joint velocities are within their joint limits, and the initial and final joint velocities and joint accelerations are both zero. From Figures 8F,G, we can see that the joint accelerations ${\ddot{\theta }}_{\text{L2}}$ and during the range 0.3s–0.5s, ${\ddot{\theta }}_{\text{L3}}$ during the range 1.6s–2s, ${\ddot{\theta }}_{\text{R3}}$ and ${\ddot{\theta }}_{\text{R5}}$ during the range 0.3s–1.3s increase sharply and are constrained by the upper and lower acceleration limits. This means that all the joint variables are in safe motion ranges. End-effector position errors ϵ of the dual-redundant-manipulators are shown in Figures 8H,I, which are very small ( ≤ 3 × 10^−4^ m). It is worth pointing out that the end-effector position errors tend to convergence as the task execution due to the position and velocity feedbacks considered in the NDSO scheme. Table 4 shows that the small joint displacements of NDSO scheme are all less than 2.4 × 10^−3^ rads.

**Table 4:** Joint drifts during the period of number “47” writing synthesized by the proposed NDSO scheme (39)–(43) which considers repetitive motion planning, joint limits, and feedback.

	**Joint displacements (rad)**	**Joint displacements (degree)**
**LEFT ARM**		
θ_L1_(2)−θ_L1_(0)	+1.78619 × 10^−3^	+0.10234
θ_L2_(2)−θ_L2_(0)	+1.40074 × 10^−4^	+0.00803
θ_L3_(2)−θ_L3_(0)	−3.31570 × 10^−4^	−0.01900
θ_L4_(2)−θ_L4_(0)	+7.76503 × 10^−5^	+0.00445
θ_L5_(2)−θ_L5_(0)	−2.15183 × 10^−3^	−0.12329
θ_L6_(2)−θ_L6_(0)	−1.79191 × 10^−3^	−0.10267
θ_L7_(2)−θ_L7_(0)	0.00000	0.00000
**RIGHT ARM**		
θ_R1_(2)−θ_R1_(0)	−1.30848 × 10^−3^	−0.07497
θ_R2_(2)−θ_R2_(0)	+4.72086 × 10^−5^	+0.00270
θ_R3_(2)−θ_R3_(0)	+2.97110 × 10^−4^	+0.01702
θ_R4_(2)−θ_R4_(0)	+2.60082 × 10^−5^	+0.00149
θ_R5_(2)−θ_R5_(0)	+2.39140 × 10^−3^	+0.13702
θ_R6_(2)−θ_R6_(0)	−6.03425 × 10^−4^	−0.03457
θ_R7_(2)−θ_R7_(0)	0.00000	0.00000

This number writing simulations further verify the effectiveness of the proposed NDSO scheme.

### 4.3. Coupled task tracking example

In order to further verify the well-coordinated performance between dual-redundant-manipulators of the proposed NDSO scheme (39)–(43), a complex end-effector-coupled task, i.e., the left arm is drawing an outside pentagram while the right arm is drawing an inside one synchronously by the dual PA10 robot manipulators. Initial joint angles of the left arm are θ_L_(0) = [0; −π/6; 0; 3π/4; 0; −π/4; 0] rad, and initial joint angles of the right arm are θ_R_(0) = [−π; −π/6; 0; 3π/4; 0; −π/4; 0] rad. The relation of the left and right end-effector tasks is

$$\left\{ \begin{array}{l} {\ddot{r}}_{\text{RX}}={\ddot{r}}_{\text{LX}}\\ {\ddot{r}}_{\text{RY}}=0.5\times {\ddot{r}}_{\text{LY}},\forall t\in \{0,T\}\\ {\ddot{r}}_{\text{RZ}}=0.5\times {\ddot{r}}_{\text{LZ}} \end{array}\right.$$

**Figure 9:**
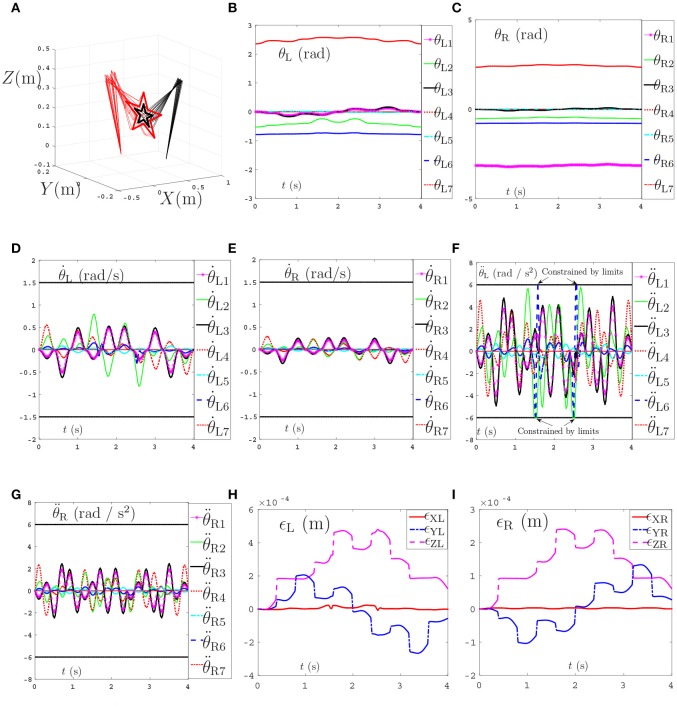
Tracking trajectories, joint angles, joint velocities, and joint accelerations during the period of end-effector-coupled pentagram-path tracking synthesized by the proposed NDSO scheme (39)–(43) which considers repetitive motion planning, joint limits, and feedbacks. **(A)** 3-D simulation tracking trajectories. **(B)** Left arm joint angle θ_L_. **(C)** Right arm joint angle θ_R_. **(D)** Left arm joint velocity ${\dot{\theta }}_{\text{L}}$. **(E)** Right arm joint velocity ${\dot{\theta }}_{\text{R}}$. **(F)** Left arm joint acceleration ${\ddot{\theta }}_{\text{L}}$. **(G)** Right arm joint acceleration ${\ddot{\theta }}_{\text{R}}$. **(H)** Position error of left arm ϵ_L_. **(I)** Position error of right arm ϵ_R_.

In the simulations, ρ_P_ and ρ_V_ in Equations (48) and (49) are set as 1 and 200 respectively. The task execution period is 4s. The tracking trajectories, joint angles, joint velocities, joint accelerations and end-effector position errors are shown in Figure 9. From Figure 9A we can see that the coupled end-effector task is completed by the dual-redundant-manipulators synthesized by NDSO scheme. What's more, the final states perfectly coincide the initial states. In addition, in Figures 9B–E, all joint angles and joint velocities are within their joint limits, and the initial and final joint velocities and joint accelerations are both zero. From Figures 9F,G, we can see that the joint accelerations ${\ddot{\theta }}_{\text{L2}}$ and ${\ddot{\theta }}_{\text{L6}}$ during 1–3s, change sharply but both are constrained by their acceleration limits. This means that all the joint variables are in the safe motion ranges. The end-effector position errors ϵ shown in Figures 9H,I are very small ( ≤ 6 × 10^−4^ m) and convergent.

In summary, the above three end-effector tasks and comparisons, i.e., pentagram-path tracking, number “47” writing, and the coupled task tracking example, demonstrate that complex end-effector tasks can be well performed by the presented NDSO scheme (39)–(43). From the simulations, it is known that the NDSO scheme can achieve the repetitive motion effectively and accurately. In addition, the position errors of the end-effectors can converge to nearly zero at the end of each cycle due to taking feedback into consideration.

### 4.4. Compared with pseudo-inverse method

In order to further illustrate the advantages of the proposed NDSO scheme, both of the traditional pseudo-inverse method and the proposed NDSO are used to perform on a dual-redundant-manipulators to track the previous coupled pentagram paths. Initial joint angles of the left and right arms are set the same as before. The formulation of the pseudo-inverse method is

(63) $$\ddot{\theta }=J^{+}{\ddot{r}}_{\text{af}}(t)-[I-J^{+}J]b(t)$$

where $\ddot{\theta }$, ${\ddot{r}}_{\text{af}}\left(t\right)$, *J* and *b*(*t*) have the same definition in the NDSO scheme. *J*^+^ means the pseudo-inverse matrix of Jacobian matrix *J* and *I* is an identity matrix in *m*+*n* dimensions.

**Figure 10:**
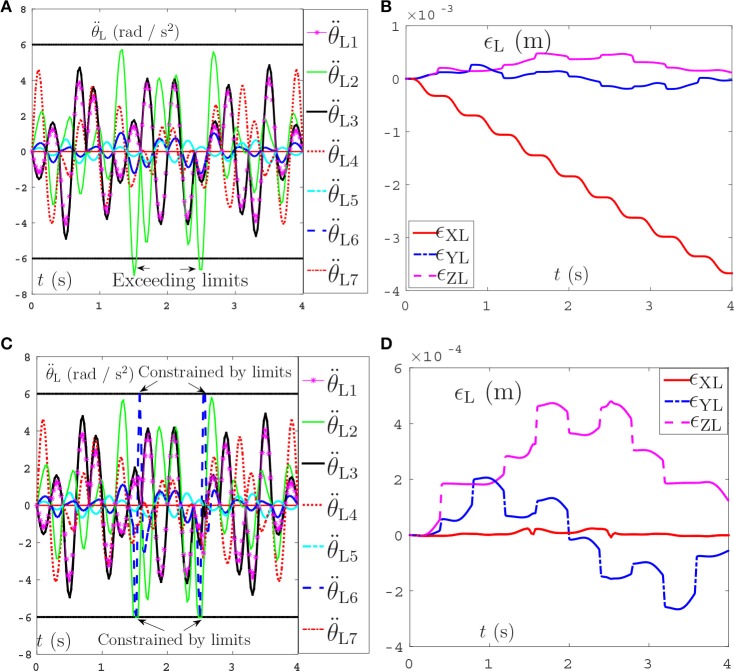
Joint accelerations and position errors of the left arm during the period of end-effector-coupled pentagram-path tracking synthesized by pseudo-inverse scheme (63) and NDSO scheme (53)–(55). **(A)** Left arm joint acceleration ${\ddot{\theta }}_{\text{L}}$ of pseudo-inverse scheme. **(B)** Position error of left arm ϵ_L_ of pseudo-inverse scheme. **(C)** Left arm joint acceleration ${\ddot{\theta }}_{\text{L}}$ of NDSO scheme. **(D)** Position error of left arm ϵ_L_ of NDSO scheme.

The comparative simulations are shown in Figure 10. Due to space limitation, only the joint acceleration and the position errors of left manipulators between the proposed NDSO scheme and the pseudo-inverse method are shown here. Specifically, Figures 10A,B show the simulation result of the pseudo-inverse method, and Figures 10C,D show the simulation result of the proposed NDSO method. From Figure 10A, we can see that the joint acceleration ${\ddot{\theta }}_{\text{L2}}$ exceeds its limits about 1.3s and 2.6s, and the end-effector position errors of the left arm shown in Figure 10C ϵ_XL_ are divergent as time goes on. That is to say, the end-effector of the dual-redundant-manipulators synthesized by the pseudo-inverse method can track the desired path but may lead to exceeding limit problem and the positioning errors will accumulate.

This comparison result further illustrate the efficiency and excellent advantages of the proposed NDSO scheme.

### 4.5. Robustness verification

**Figure 11:**
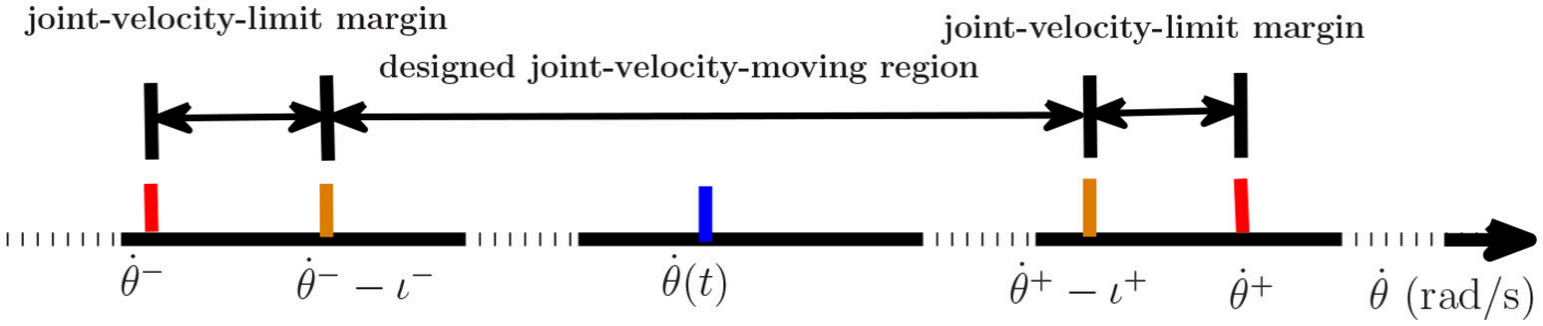
The form of the joint-velocity-limit margins and joint-velocity moving region where ι^−^ is minus and ι^+^ is positive.

In this subsection, systematic errors are taken into consideration and the perturbed LVI-PDNN in Equation (62) is used to solve the path-tracking problem of the dual redundant manipulators. The pentagram path-tracking task in 4.1 is adopted to compare the joint displacements without perturbation in Table 3. During the simulations, error-matrix Δ*D* and error-vector Δ*S* are generated randomly. The element Δ_*i*_ of them is formulated as

(64) $$\Delta_{i}=0.1*\nu_{a}(\nu_{c}sin(\nu_{b}t)+(1-\nu_{c})cos(\nu_{b}t))$$

where ν_*a*_ is a random integer in [−5, 5], ν_*b*_ is a random integer in [1, 5] and ν_*c*_ is a random integer in [0, 1]. All of them are distributed evenly. ν_*a*_ and ν_*b*_ control the amplitude and frequency of the element respectively. ν_*c*_ controls the form of the perturbation function to be sine function (ν_*c*_ = 1) or to be cosine function (ν_*c*_ = 0). The initial joint angles of dual arms are set as same in 4.1. The parameters $d,x^{-},x^{+},\rho_{\text{p}},\rho_{\text{v}}$ are set according to the 4th set of equations in Table 2. Inspired by Zhang and Zhang (2013b), we consider joint-velocity-limit margins ι shown in Figure 11 in our experiments. The updated ${\dot{\theta }}_{\text{L}}^{\pm }\left(t\right)$ and ${\dot{\theta }}_{\text{R}}^{\pm }\left(t\right)$ in (51) are shown in Table 5, where the margins considered joint-velocity-limits are highlighted in bold.

**Table 5:** ${\dot{\theta }}_{\text{L}}^{\pm }$ and ${\dot{\theta }}_{\text{R}}^{\pm }$ (rad/s) used in Robustness experiments.

	**Joint L1**	**Joint L2**	**Joint L3**	**Joint L4**	**Joint L5**	**Joint L6**	**Joint L7**
**LEFT ARM**		
${\dot{\theta }}^{+}$	1.5	1.5	1.5	1.5	1.5	1.5	1.5
${\dot{\theta }}^{-}$	−**0.5**	−1.5	−1.5	−1.5	**-0.5**	**-0.5**	−1.5
**RIGHT ARM**		
${\dot{\theta }}^{+}$	**0.5**	1.5	1.5	1.5	**0.4**	1.5	1.5
${\dot{\theta }}^{-}$	−1.5	−1.5	−1.5	−1.5	−1.5	**-0.4**	−1.5

**Figure 12:**
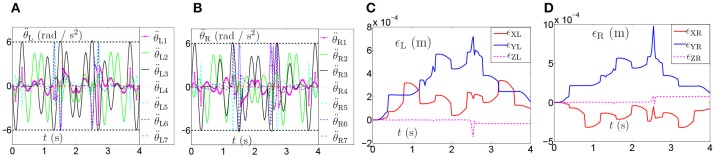
Joint accelerations and position errors during the period of pentagram-path tracking task synthesized by NDSO scheme considering differentiation errors and implementation errors. **(A)** Left arm joint acceleration ${\ddot{\theta }}_{\text{L}}$; **(B)** Right arm joint acceleration ${\ddot{\theta }}_{\text{R}}$; **(C)** Position error of left arm ϵ_L_; **(D)** Position error of right arm ϵ_R_.

The joint drifts of dual arms are shown in Table 6, which shows that the joint displacement of every joint almost has no change compared with the result in Table 3. The joint accelerations and position errors during the period of pentagram-path tracking task are recorded in Figures 12A–D. The curves in Figures 12A–D show that the joint accelerations are all constrained within the limits (i.e., ±6rad/*s*^2^). Besides, the position errors have been controlled within a very small range which is lower than 1 × 10^−3^ (m). Although there exists time-varying systematic perturbation, the position errors are still convergent at the end of the task execution. In summary, the proposed NDSO method performs well under the perturbation and has strong robustness.

**Table 6:** Joint drifts when dual-redundant-manipulators tracking a pentagram-path synthesized by NDSO scheme considering differentiation errors and implementation errors.

	**Joint displacements (rad)**	**Joint displacements (degree)**
**LEFT ARM**		
θ_L1_(4) − θ_L1_(0)	+3.89070 × 10^−3^	+0.22292
θ_L2_(4) − θ_L2_(0)	+1.86462 × 10^−4^	+0.01068
θ_L3_(4) − θ_L3_(0)	−8.38750 × 10^−4^	−0.04806
θ_L4_(4) − θ_L4_(0)	−3.11366 × 10^−5^	−0.00178
θ_L5_(4) − θ_L5_(0)	−5.90065 × 10^−3^	−0.33808
θ_L6_(4) − θ_L6_(0)	−1.51023 × 10^−3^	−0.08653
θ_L7_(4) − θ_L7_(0)	−6.41855 × 10^−6^	−0.00037
**RIGHT ARM**		
θ_R1_(4) − θ_R1_(0)	−3.54920 × 10^−3^	−0.20335
θ_R2_(4) − θ_R2_(0)	−1.56336 × 10^−5^	−0.00090
θ_R3_(4) − θ_R3_(0)	+7.25769 × 10^−4^	+0.04158
θ_R4_(4) − θ_R4_(0)	+2.49020 × 10^−4^	+0.01427
θ_R5_(4) − θ_R5_(0)	+5.66373 × 10^−3^	+0.32451
θ_R6_(4) − θ_R6_(0)	−8.71307 × 10^−4^	−0.04992
θ_R7_(4) − θ_R7_(0)	+1.68829 × 10^−5^	+0.00097

## 5. Conclusion

In this paper, a neural-dynamic based synchronous-optimization scheme of dual redundant robot manipulators scheme (NDSO) of dual-redundant-manipulators for tracking complex paths has been proposed to solve the joint-drift problem. The scheme can not only finish the end-effector task collaboratively with the dual-redundant-manipulators, but also achieve repetitive motion, avoid physical limits and position-error convergence. First, the left and right manipulator subschemes are formulated and then are combined to one quadratic program scheme, i.e., the NDSO scheme. Next, the scheme is unified into a standard quadratic programming problem. Finally, the quadratic programming problem is solved by a linear-variational-inequality primal-dual neural networks. Three complex end-effector tasks and comparisons, i.e., pentagram-path tracking, number writing, and coupled tasks have verified the effectiveness, accuracy, repeatability, safety, generality and robustness of the proposed NDSO scheme. To the best of authors' knowledge, it is the first time to propose such a NDSO scheme with so many optimization criteria and can solve the joint-drift problems in three three-dimensional workspace. The future work is to exploit higher efficient resolving algorithms to further improve the performance of the scheme and consider the control input saturation problem and uncertainties.

## Author contributions

The idea of the paper that we can try to solve the optimization problem on the acceleration level of dual redundant manipulators is proposed by ZZ. The paper is drafted and revised by ZZ and QZ together. QZ and WF design and implement the experiment in coordination.

### Conflict of interest statement

The authors declare that the research was conducted in the absence of any commercial or financial relationships that could be construed as a potential conflict of interest.

## References

[B1] Cai B. Zhang Y. (2012). Different-level redundancy-resolution and its equivalent relationship analysis for robot manipulators using gradient-descent and zhang 's neural-dynamic methods. IEEE Trans. Indus. Electron. 59, 3146–3155. 10.1109/TIE.2011.2106092

[B2] Cheng F. T. Chen T. H. Sun Y. Y. (1994). Resolving manipulator redundancy under inequality constraints. IEEE Trans. Robot. Autom. 10, 65–71. 10.1109/70.285587

[B3] Chevallereau C. Khalil W. (1988). “A new method for the solution of the inverse kinematics of redundant robots,” in Proceedings of IEEE International Conference on Robotics and Automation, Vol. 1 (Philadelphia, PA), 37–42.

[B4] Chikhaoui M. T. Granna J. Starke J. Burgner-Kahrs J. (2018). Toward motion coordination control and design optimization for dual-arm concentric tube continuum robots. IEEE Robot. Autom. Lett. 3, 1793–1800. 10.1109/LRA.2018.2800037

[B5] Dong I. P. Kim H. Park C. Kim D. (2017). “Design and analysis of the dual arm manipulator for rescue robot,” in Proceedings of IEEE International Conference on Advanced Intelligent Mechatronics, Vol. 1 (Munich), 608–612.

[B6] Eremin E. L. Shelenok E. A. (2017). “Simulation modeling of the control system for robotic manipulator with input saturation,” in Proceedings of International Siberian Conference on Control and Communications (Astana), 1–5.

[B7] Felip J. Morales A. (2015). “A solution for the cap unscrewing task with a dual arm sensor-based system,” in Proceedings of the 15th IEEE-RAS International Conference on Humanoid Robots, Vol. 1 (Seoul), 823–828.

[B8] Flacco F. De Luca A. (2015). Discrete-time redundancy resolution at the velocity level with acceleration/torque optimization properties. Robot. Auton. Syst. 70, 191–201. 10.1016/j.robot.2015.02.008

[B9] Guo D. Su Z. Sun S. Lin X. Liu Q. (2017). “A new feedback-added obstacle avoidance scheme for motion planning of redundant robot manipulators,” in Proceedings of the 29th Chinese Control and Decision Conference, Vol. 1 (Chongqing), 6601–6606.

[B10] Hartman and Philip (1982). Ordinary Differential Equations, 2nd Edn. Boston, MA: Birkhauser.

[B11] Ho E. S. L. Komura T. Lau R. W. H. (2005). Computing inverse kinematics with linear programming. in ACM Symposium on Virtual Reality Software and Technology, Vol. 1 (Monterey, CA), 163–166.

[B12] Huang S. Xiang J. Wei W. Chen M. Z. Q. (2017). On the virtual joints for kinematic control of redundant manipulators with multiple constraints. IEEE Trans. Control Sys. Technol. 26, 65–76. 10.1109/TCST.2017.2650684

[B13] Jin L. Li S. (2016). Distributed task allocation of multiple robots: A control perspective. IEEE Trans. Sys. Man Cybern. Sys. 48, 693–701. 10.1109/TSMC.2016.2627579

[B14] Jin L. Liao B. Liu M. Xiao L. Guo D. Yan X. (2017). Different-level simultaneous minimization scheme for fault tolerance of redundant manipulator aided with discrete-time recurrent neural network. Front. Neurorobot. 11, 1–7. 10.3389/fnbot.2017.00050 28955217 PMC5601992

[B15] Jin L. Zhang Y. (2014). G2-type srmpc scheme for synchronous manipulation of two redundant robot arms. IEEE Trans. Cybern. 45, 153–164. 10.1109/TCYB.2014.2321390 24846689

[B16] Jr R. S. J. Roberts R. G. (2015). A more compact expression of relative jacobian based on individual manipulator jacobians. Robot. Auton. Sys. 63, 158–164. 10.1016/j.robot.2014.08.011

[B17] Klein C. A. Kee K. B. (1989). The nature of drift in pseudoinverse control of kinematically redundant manipulators. IEEE Trans. Robot. Autom. 5, 231–234. 10.1109/70.88043

[B18] Lee J. Chang P. H. Jamisola R. S. (2014). Relative impedance control for dual-arm robots performing asymmetric bimanual tasks. IEEE Trans. Indus. Electron. 61, 3786–3796. 10.1109/TIE.2013.2266079

[B19] Lin X. Zhang Y. (2013). Acceleration-level repetitive motion planning and its experimental verification on a six-link planar robot manipulator. IEEE Trans. Control Sys. Technol. 21, 906–914. 10.1109/TCST.2012.2190142

[B20] Liu Z. Chen C. Zhang Y. Chen C. L. (2015). Adaptive neural control for dual-arm coordination of humanoid robot with unknown nonlinearities in output mechanism. IEEE Trans. Cybern. 45, 507–518. 10.1109/TCYB.2014.2329931 24968367

[B21] Luo J. Zhang J. Xie Z. Zhang X. Xiao L. Su X. (2017). “Acceleration-level inverse-free g2 scheme for inverse kinematics path tracking of robot manipulators,” in Proceedings of the 36th Chinese Control Conference, Vol. 1 (Dalian), 6804–6809.

[B22] Reynoso-Mora P. Chen W. Tomizuka M. (2016). A convex relaxation for the time-optimal trajectory planning of robotic manipulators along predetermined geometric paths. Opt. Control Applic. Methods 37, 1263–1281. 10.1002/oca.2234

[B23] Shin S. Kim C. (2015). Human-like motion generation and control for humanoid's dual arm object manipulation. IEEE Trans. Indus. Electron. 62, 2265–2276. 10.1109/TIE.2014.2353017

[B24] Sun N. Fang Y. Chen H. Fu Y. Lu B. (2018). Nonlinear stabilizing control for ship-mounted cranes with ship roll and heave movements: design, analysis, and experiments. IEEE Trans. Sys. Man Cybern. Sys. 48, 1–13. 10.1109/TSMC.2017.2700393

[B25] Sun N. Fang Y. Chen H. Lu B. (2017). Amplitude-saturated nonlinear output feedback antiswing control for underactuated cranes with double-pendulum cargo dynamics. IEEE Trans. Indus. Electron. 64, 2135–2146. 10.1109/TIE.2016.2623258

[B26] Tewari A. (2002). Modern Control Design With MATLAB and SIMULINK[M]. New Jersey, NJ: John Wiley and Sons, Ltd.

[B27] Toshani H. Farrokhi M. (2014). Real-time inverse kinematics of redundant manipulators using neural networks and quadratic programming: a lyapunov-based approach. Robot. Auton. Sys. 62, 766–781. 10.1016/j.robot.2014.02.005

[B28] Tran T. T. Ge S. S. He W. (2015). “Adaptive control for a robotic manipulator with uncertainties and input saturations,” in Proceedings of IEEE International Conference on Mechatronics and Automation, Vol. 1, 1525–1530.

[B29] Wang Y. Yan X. He L. Tan H. Zhang Y. (2015). “Inverse-free solution of z1g1 type to acceleration-level inverse kinematics of redundant robot manipulators,” in Proceedings of the 7th International Conference on Advanced Computational Intelligence, Vol. 1 (Mount Wuyi), 57–62.

[B30] Xiao Y. Fan Z. Li W. Chen S. Zhao L. Xie H. (2016). “A manipulator design optimization based on constrained multi-objective evolutionary algorithms,” in Proceedings of International Conference on Industrial Informatics - Computing Technology, Intelligent Technology, Industrial Information Integration, Vol. 1 (Wuhan), 199–205.

[B31] Zhang P. Yan Z. Wang J. (2014). “Obstacle and singularity avoidance for kinematically redundant manipulators based on neurodynamic optimization,.” in Proceedings of the 5th International Conference on Intelligent Control and Information Processing, Vol. 1 (Dalian), 460–465.

[B32] Zhang Y. Ge S. S. Lee T. H. (2004). A unified quadratic-programming-based dynamical system approach to joint torque optimization of physically constrained redundant manipulators. IEEE Trans. Sys. Man Cybern. B 34, 2126–2132. 10.1109/TSMCB.2004.830347 15503508

[B33] Zhang Y. Li S. Gui J. Luo X. (2018). Velocity-level control with compliance to acceleration-level constraints: a novel scheme for manipulator redundancy resolution. IEEE Trans. Indus. Inform. 14, 921–930. 10.1109/TII.2017.2737363

[B34] Zhang Y. Lv X. Li Z. Yang Z. Chen K. (2008). Repetitive motion planning of pa10 robot arm subject to joint physical limits and using lvi-based primalcdual neural network. Mechatronics 18, 475–485. 10.1016/j.mechatronics.2008.04.005

[B35] Zhang Y. Zhang Z. (2013a). Repetitive Motion Planning and Control of Redundant Robot Manipulators. Berlin: Heidelberg: Springer.

[B36] Zhang Z. Li Z. Zhang Y. Luo Y. Li Y. (2015). Neural-dynamic-method-based dual-arm cmg scheme with time-varying constraints applied to humanoid robots. IEEE Trans. Neural Netw. Learn. Sys. 26, 3251–3262. 10.1109/TNNLS.2015.2469147 26340789

[B37] Zhang Z. Zhang Y. (2012). Acceleration-level cyclic-motion generation of constrained redundant robots tracking different paths. IEEE Trans. Sys. Man Cybern. B 42, 1257–1269. 10.1109/TSMCB.2012.2189003 22481829

[B38] Zhang Z. Zhang Y. (2013b). Repetitive motion planning and control on redundant robot manipulators with push-rod-type joints. J. Dyn. Sys. Measur. Control 135, 44–45. 10.1115/1.4007608

[B39] Zheng Y. Luh J. (1986). “Joint torques for control of two coordinated moving robots,” in Proceedings of IEEE International Conference on Robotics and Automation (San Francisco, CA), 1375–1380.

